# Effectiveness and safety of topical levodopa in a chick model of myopia

**DOI:** 10.1038/s41598-019-54789-5

**Published:** 2019-12-04

**Authors:** Kate Thomson, Cindy Karouta, Ian Morgan, Tamsin Kelly, Regan Ashby

**Affiliations:** 1 Centre for Research into Therapeutic Solutions, Faculty of Science and Technology, University of Canberra, Canberra, Australia; 2 Research School of Biology, Australian National University, Canberra, Australia; 3 National Centre for Forensic Studies, Faculty of Science and Technology, University of Canberra, Canberra, Australia

**Keywords:** Preclinical research, Retina

## Abstract

Animal models have demonstrated a link between dysregulation of the retinal dopamine system and the excessive ocular growth associated with the development of myopia. Here we show that intravitreal or topical application of levodopa, which is widely used in the treatment of neurological disorders involving dysregulation of the dopaminergic system, inhibits the development of experimental myopia in chickens. Levodopa slows ocular growth in a dose dependent manner in chicks with a similar potency to atropine, a common inhibitor of ocular growth in humans. Topical levodopa remains effective over chronic treatment periods, with its effectiveness enhanced by coadministration with carbidopa to prevent its premature metabolism. No changes in normal ocular development (biometry and refraction), retinal health (histology), or intraocular pressure were observed in response to chronic treatment (4 weeks). With a focus on possible clinical use in humans, translation of these avian safety findings to a mammalian model (mouse) illustrate that chronic levodopa treatment (9 months) does not induce any observable changes in visual function (electroretinogram recordings), ocular development, and retinal health, suggesting that levodopa may have potential as a therapeutic intervention for human myopia.

## Introduction

Myopia, or short-sightedness, is a refractive disorder arising from a mismatch between the optical power of the eye and its axial length. This is due to excessive elongation of the eye during development and into young adulthood which leads to the focal plane of distant objects falling in front of the retina, instead of on it, causing the image to appear blurred. Myopia is now recognised as a leading cause of visual impairment and low vision world-wide^1^. Over the past 50 years, myopia rates have increased dramatically, with some estimates predicting that half of the world’s population could be affected by short-sightedness by 2050^1^. The rapid rise in myopia prevalence is most evident in educationally developed areas of East and Southeast Asia (for review see^2^). In these locations, the prevalence of myopia in young adults has risen from 20–30% to 80–85% over one generation^2,3^. Over the same period, the prevalence of high myopia has increased from 1–2% to 10–20%^2^.

These changes in prevalence pose two main challenges. The first is the need to provide optical or other corrections for the associated refractive error for a large percentage of the population. Arguably an even greater challenge comes from the increased prevalence of high myopia, and its associated sight-threatening pathological changes^4^. Correction of the refractive error does not prevent the development of these conditions, the chances of which increase with the severity of myopia, as it does not address the excessive elongation of the eye^4^. Such pathologies include chorio-retinal changes including retinal detachments, myopic macular degeneration, and staphyloma, as well as an increased risk of other sight-threatening conditions such as glaucoma and cataracts (for review see^5^). The US National Eye Institute has estimated the annual cost for treating refractive disorders in the US alone at just under $14 billion in 2010 and rising^6^, with considerable indirect costs such as lost productivity unaccounted for. The social returns to be obtained through myopia prevention are therefore significant. For this reason, a deeper understanding of how ocular growth is regulated, and the development of preventive interventions to slow the onset or progression of myopia, are urgently needed.

Animal studies have demonstrated that ocular growth is regulated locally in response to visual stimuli by pathways originating in the retina, with the retinal transmitter dopamine playing a key role (for review see^7,8^). Dopamine release is strongly affected by light and the spatiotemporal properties of visual inputs, with dysregulation of the dopaminergic system heavily implicated in the development of experimental myopia (for review see^7,8^). Specifically, in multiple species, retinal dopamine synthesis and release has been shown to be significantly down-regulated during the development of experimental myopia^9–13^, whilst pharmacological administration of dopamine agonists, which mimic the effects of dopamine, have been shown to inhibit the development of experimental myopia (for review see^7^). Furthermore, intravitreal administration of exogenous dopamine in rabbits^14^ and systemic administration of its precursor levodopa (L-DOPA) in guinea pigs^15^ inhibit the development of experimental myopia, while retina-specific tyrosine hydroxylase knockout mice and mice treated with 6-hydroxydopamine, which depletes the retina of dopaminergic neurons, show a myopic shift in refraction^13,16^. Finally, dopaminergic activity appears to underly the mechanism by which bright light exposure, or exposure to brief periods of normal vision, prevents the development of experimental myopia, specifically form-deprivation myopia (FDM), in the chick^17–27^.

Based on the potential role of dopamine in the regulation of ocular growth, this article investigates whether topical administration of levodopa, a drug widely used to treat neurological disorders involving dopaminergic disfunction^28^, can inhibit ocular growth and therefore prevent the development of experimental myopia in an animal model (chicken). Here we show that levodopa slows ocular growth and inhibits experimental myopia in a dose-dependent manner in chicks with a similar potency to atropine, whilst not affecting ocular health across those parameters tested following chronic administration. For translational potential, this avian safety data was further complemented in a second, mammalian, model by analysing the effects of chronic levodopa treatment on ocular safety in mice.

## Results

### Effectiveness of levodopa in preventing experimental myopia in chickens

#### Intravitreal injections inhibit form-deprivation myopia in a dose dependent manner

To investigate whether levodopa inhibits experimental myopia when introduced directly to the retina, levodopa was administered to chicks undergoing FDM as a daily intravitreal injection at one of four concentrations (0.15 mM, 1.5 mM, 15 mM, and 75 mM; Supplementary Table S-1A) for four days.

**Figure 1:**
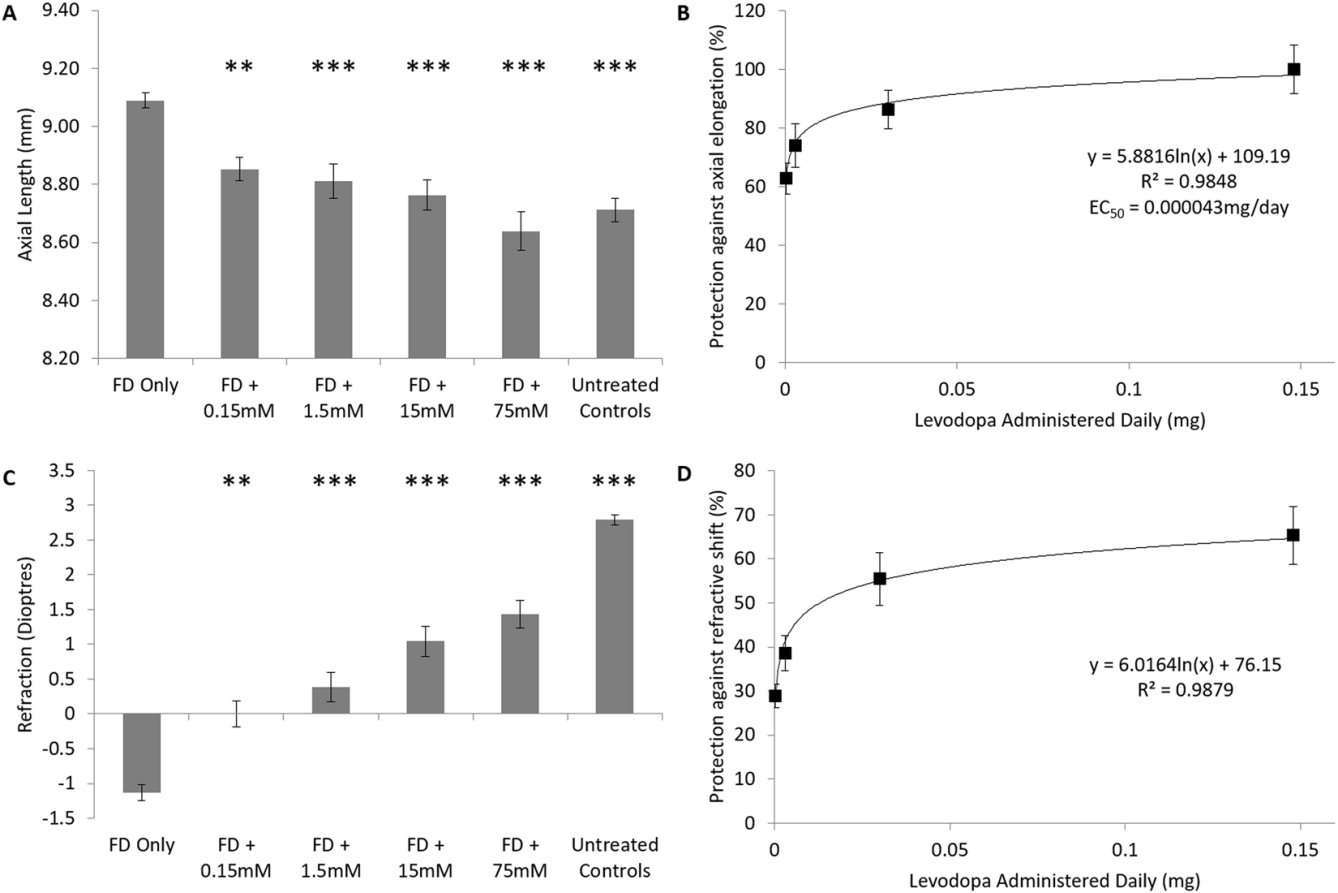
Dose Response Curve – Levodopa Intravitreal Injections. **(A)** Axial length after four days of treatment for each of the tested conditions. **(B)** Percent protection against the axial elongation associated with diffuser wear (FD) relative to levodopa concentration administered. **(C)** Refractive values after four days of treatment for each of the tested conditions. **(D)** Percent protection against the myopic refractive shift associated with FD relative to levodopa concentration administered. Data represent mean ± standard error of the mean. FD: Form deprivation. Untreated controls: age-matched untreated controls. Statistics denote difference to FD Only; *p < 0.05, **p < 0.02, ***p < 0.001.

Ocular biometry: Ocular growth in untreated contralateral control eyes was not affected by intravitreal injection of levodopa, with no significant difference in axial length observed between contralateral control and age-matched untreated control eyes following 4 days of treatment (ANOVA, F(5, 84) = 0.284, p = 0.921). As expected, form-deprivation (FD, myopia induction) induced a significantly greater rate of axial elongation in diffuser-treated eyes relative to that seen in contralateral control or age-matched untreated control eyes over the four-day treatment period (p < 0.001, Fig. 1A, Supplementary Table S-2). This excessive elongation was, however, inhibited in a dose-dependent manner by the daily intravitreal injection of levodopa (ANOVA, F(4,68) = 15.072, p < 0.001; Fig. 1A), with the protective effect best described by a logarithmic relationship (y = 5.8816In(x) + 109.19, r^2^ = 0.9848; Fig. 1B), with an EC_50_ of 0.000043 mg/day (0.0008 mM or 0.0007% w/v). All concentrations of levodopa tested induced a significant reduction in axial elongation relative to FDM alone (0.15 mM: p < 0.02; 1.5–75 mM: p < 0.001, Fig. 1A).

At all concentrations tested, levodopa did not affect anterior chamber depth (ANOVA, F(5,84) = 1.109, p = 0.361) or lens thickness (ANOVA, F(5, 84) = 0.563, p = 0.728), but instead, changes in axial length correlated solely with changes in vitreal chamber depth (ANOVA, F(5, 84) = 15.042, P < 0.001).

Daily intravitreal injection of levodopa over a four-day period did not affect normal ocular development when administered to otherwise untreated birds (p = 1.000; Supplementary Table S-2), nor did it affect contralateral control eyes (ANOVA, F(5, 84) = 0.353, p = 0.879). Furthermore, intravitreal injection of the vehicle solution did not prevent the axial elongation associated with FD (p = 1.000).

Refraction: As with axial length, refraction in untreated contralateral control eyes was also not affected by intravitreal injection of levodopa, with no significant difference observed between contralateral control and age-matched untreated control eyes at cessation of treatment (ANOVA, F(5, 84) = 0.454, p = 0.808). Form-deprivation induced a significant myopic shift in refraction relative to that seen in contralateral control and age-matched untreated control eyes (p < 0.001, Fig. 1C,D, Supplementary Table S-2). This myopic shift was inhibited in a dose-dependent manner by the daily intravitreal injection of levodopa (y = 6.0164In(x) + 76.15, r^2^ = 0.9879); Fig. 1D), with greater protection seen with increasing concentrations (Fig. 1C, ANOVA, F(4,68) = 21.654, p < 0.001). At all concentrations, treated eyes showed a less myopic refraction compared to that observed in FD only chickens (0.15 mM: p < 0.02, 1.5–75 mM: p < 0.001; Fig. 1C), however, all levodopa groups still showed a relative myopic shift when compared to age-matched untreated control animals (p < 0.001 in all groups; Fig. 1C).

As with axial length, there was no effect on refractive development in contralateral control eyes (Supplementary Table S-2; ANOVA, F(5, 84) = 0.665, p = 0.651). Similarly, daily intravitreal injection of levodopa did not affect normal refractive development when administered to otherwise untreated eyes (p = 0.128). Intravitreal injection of the vehicle solution alone did not alter myopic refractive development in FD eyes compared to FD only chicks (p = 0.093).

#### Topical eye drops inhibit form-deprivation myopia in a dose dependent manner

To examine whether levodopa is effective at inhibiting FDM when applied topically to the eye, as it is for intravitreal injections, twice-daily eye drops of levodopa were administered to chicks undergoing FD at one of four concentrations (0.15 mM, 1.5 mM, 15 mM, and 45 mM; Supplementary Table S-1B) for four days.

**Figure 2:**
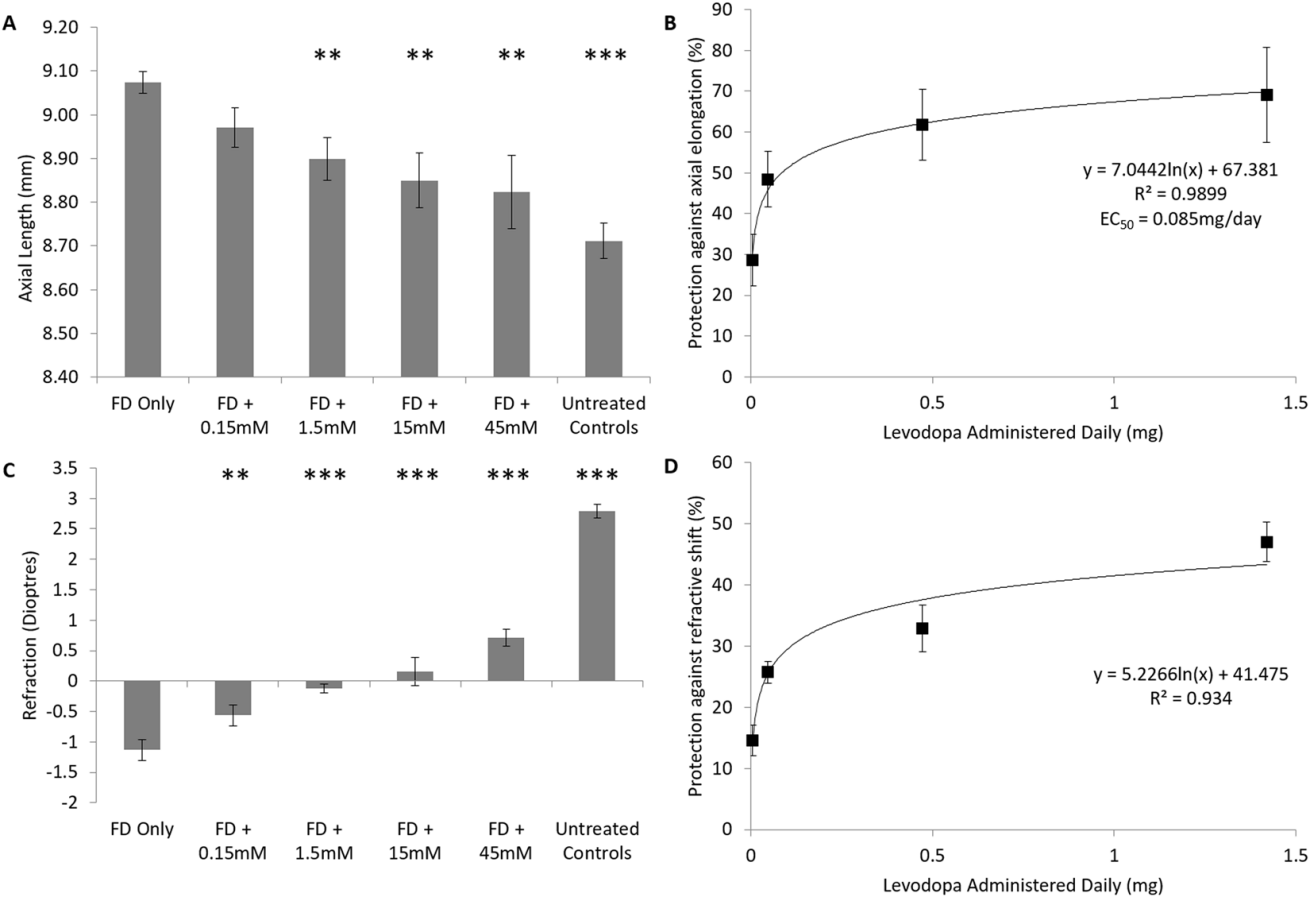
Dose Response Curve – Levodopa Topical Eye Drops. **(A)** Axial length after four days of treatment for each tested condition. **(B)** Percent protection against the axial elongation associated with form-deprivation (FD) relative to amount of levodopa administered. **(C)** Refractive values after four days of treatment for each tested condition. **(D)** Percent protection against the myopic refractive shift associated with FD relative to amount of levodopa administered. Data represents mean ± standard error of the means FD: Form deprivation. Untreated controls: age-matched untreated controls. Statistics denote difference to FD Only; *p < 0.05, **p < 0.02, ***p < 0.001.

Ocular biometry: As with intravitreal injections, ocular growth in contralateral control eyes was not altered by levodopa eye drops, with no significant difference in axial length observed between contralateral control and age-matched untreated control eyes at the end of treatment (ANOVA, F(5,101) = 0.454, p = 0.810). Twice-daily topical application of levodopa significantly inhibited the excessive axial elongation associated with myopia induction in a dose-dependent manner (Fig. 2A, Supplementary Table S-3; ANOVA, F(4,85) = 5.494, p < 0.01). This effect was best described by a logarithmic function (y = 7.0442In(x) + 67.381, r^2^ = 0.9899; Fig. 2B), with an EC_50_ of 0.085 mg/day (2.69 mM or 0.053% w/v). At the highest dose (45 mM), there was no significant difference in axial length to those values seen in age-matched untreated control eyes (p = 0.186). Like that observed for intravitreal injections, topical levodopa treatment, at all concentrations, did not affect anterior chamber depth (ANOVA, F(5,93) = 0.990, p = 0.428) or lens thickness (ANOVA, F(5,93) = 0.741, p = 0.594), but rather the changes in axial length observed were associated with changes in vitreal chamber depth (ANOVA, F(5,93) = 9.420, p < 0.001; Supplementary Table S-3). Furthermore, analysis of levodopa at 15 mM demonstrated no effects on corneal curvature (levodopa treated eyes 3.2 ± 0.6 mm radius of curvature vs contralateral control eyes 3.3 ± 0.4 mm radius of curvature; n = 5, p = 0.501) or corneal thickness (levodopa treated eyes 215.9 ± 5.3 µm vs contralateral control eyes 214.5 ± 7.5 µm; n = 5, p = 0.400).

Topical application of levodopa did not alter axial development compared to age-matched untreated controls (Supplementary Table S-3) when administered to otherwise untreated chickens (p = 1.000), nor did it affect ocular growth in contralateral control eyes (ANOVA, F(5,93) = 1.294, p = 0.273). Topical administration of the vehicle solution did not prevent or alter the excessive axial growth associated with FDM (p = 0.285), indicating that the presence of levodopa was critical in inhibiting experimental myopia.

Refraction: Consistent with the data presented thus far, levodopa eye drops did not alter the refractive development of contralateral control eyes relative to age-matched untreated control eyes (ANOVA, F(5, 101) = 1.438, p = 0.223). Topical administration of levodopa significantly inhibited the negative shift in refraction associated with FD in a dose-dependent manner (0.15 mM: p < 0.02; 1.5 mM, 15 mM, 45 mM: p < 0.001: Fig. 2C,D, Supplementary Table S-3), with the protective effect best described by a logarithmic relationship (y = 5.2266In(x) + 41.475, R^2^ = 0.934; Fig. 2D). However, as with intravitreal injections, topically treated eyes still showed a relative myopic shift relative to age-matched untreated chickens (ANOVA, F(4,64) = 149.165, p < 0.001).

Refractive development (Supplementary Table S-3) was unaffected in contralateral control eyes of animals treated with levodopa (ANOVA, F(5,93) = 0.959, p = 0.448) or in otherwise untreated eyes (p = 1.000). Eye drops containing only the vehicle solution did not alter the degree of myopic shift in diffuser-treated eyes (p = 1.000). This once again indicates that levodopa, while not altering normal refractive development, was the factor responsible for inhibiting the myopic refractive changes generated by FD.

#### Modification of bioavailability enhances the potency of topical eye drops

To investigate whether the protective effects observed with topical application of levodopa could be enhanced by increasing bioavailability, levodopa eye drops were administered in the presence of carbidopa (4:1 levodopa:carbidopa ratio, to prevent premature metabolism), or 10% dimethyl sulfoxide (DMSO, to increase corneal penetration) for a period of four days (Supplementary Table S-1B).

**Figure 3:**
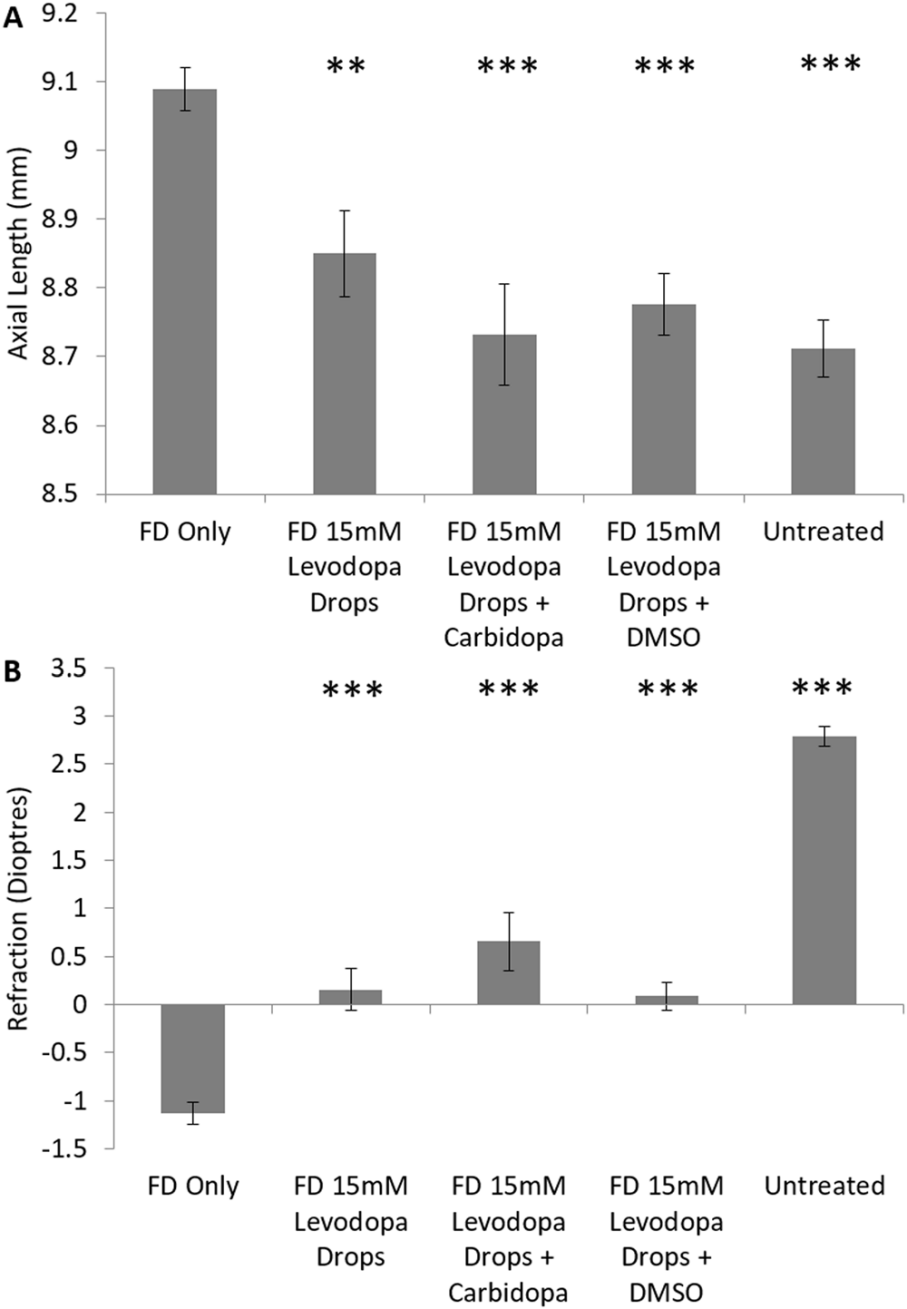
Topical Eye Drops – Modification of Bioavailability. **(A)** Axial length measurements following four days of treatment for each tested condition. **(B)** Refraction following four days of treatment for each tested condition. Data represents mean ± standard error of the mean. FD: Form deprivation. Untreated controls: age-matched untreated controls. DMSO: Dimethyl sulfoxide. Statistics denote difference to FD Only; *p < 0.05, **p < 0.02, ***p < 0.001.

The addition of carbidopa, at a 1:4 ratio to levodopa (3.75 mM carbidopa:15 mM levodopa), significantly enhanced the protective effects observed relative to levodopa alone against FD for both axial length (94.6% ± 9.73 protection, p < 0.001; 32.8% increase from levodopa alone, p < 0.02; Fig. 3A, Supplementary Table S-3) and refraction (45.6% ± 6.89 protection, p < 0.001; 12.8% increase from levodopa alone, p < 0.001; Fig. 3B, Supplementary Table S-3) over a four day period of treatment.

Those animals treated with a levodopa/DMSO solution demonstrated slightly shorter axial lengths than those animals treated with levodopa alone (83.1% ± 8.39 protection, p < 0.001; 21.3% increase from levodopa alone, p < 0.02; Fig. 3A, Supplementary Table S-3). However, there was no significant difference in the degree to which the myopic refractive shift was lessened between levodopa and levodopa/DMSO treated animals (Fig. 3B, Supplementary Table S-3).

#### Levodopa remains effective over chronic treatment periods

To examine whether the protective effects of topical levodopa can be maintained over chronic treatment periods, levodopa, levodopa/carbidopa, and levodopa/0.1% benzalkonium chloride (BAK, a frequently used ophthalmic alternative to DMSO) solutions were topically administered (Supplementary Table S-1B) to chicks undergoing FD for four weeks (approximately one third of the ocular maturation period of chicks). At the end of the treatment period ocular samples were examined to assess the retina for changes in architecture or signs of apoptosis.

**Figure 4:**
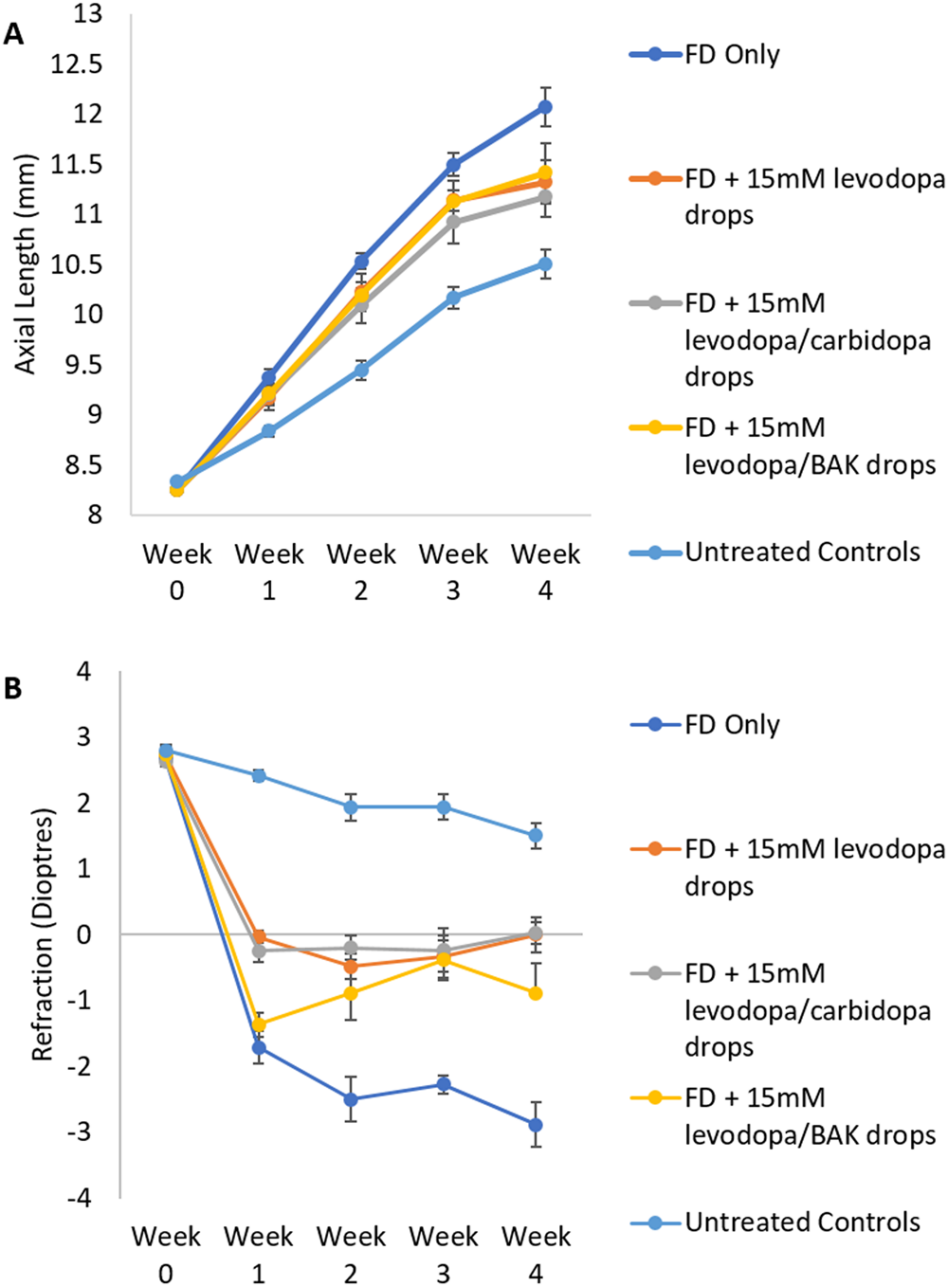
Long-term effectiveness of 15 mM levodopa drops. **(A)** Weekly axial length measurements for form deprived, drug treated eyes. **(B)** Weekly refraction for form deprived, drug treated eyes. Data represents mean ± standard error of the mean. BAK: Benzalkonium chloride. FD: Form deprivation. Untreated controls: age-matched untreated controls.

The excessive axial elongation associated with FD was significantly inhibited by levodopa treatment over 4 weeks compared to FD-only animals (Wilks’ Lambda = 0.425, F(3,32) = 1.816, p < 0.05; Supplementary Table S-4; Fig. 4A; 56.9% ± 1.09 protection). This protective effect was enhanced by the addition of carbidopa (levodopa: p < 0.01, to levodopa/carbidopa (65.1% ± 1.19 protection): p < 0.001, at week 4, Fig. 4A), but was no more protective with the addition of 0.1% w/v BAK (levodopa: p < 0.01, to levodopa/BAK (51.62% ± 1.31 protection) p = 0.14, Fig. 4A). Levodopa, levodopa/carbidopa, or levodopa/BAK did not alter anterior chamber depth (Wilks’ Lambda = 0.602, F(3, 32) = 0.938, p = 0.517) or lens thickness (Wilks’ Lambda = 0.579, F(3, 32) = 1.638, p = 0.126) over 4 weeks of treatment (Supplementary Table S-4). Hence all ocular changes observed arose through changes in vitreal chamber depth (Wilks’ Lambda = 0.453, F(3,32) = 1.911, p < 0.05).

In accordance with axial length changes, long-term topical treatment significantly inhibited the myopic shift in refraction seen in response to FD (Wilks’ Lambda = 0.078, F(3,32) = 7.569, p < 0.001; Supplementary Table S-5; Fig. 4B), with all treated animals exhibiting significantly less myopic refractions than FD-only treated animals at week 4 (all groups: p < 0.001), and no significant difference between treatment groups (levodopa (65.6% ± 5.36 protection) vs levodopa:carbidopa (66.4% ± 8.36 protection): p = 1.000; levodopa vs levodopa/BAK (45.5% ± 4.03 protection): p = 0.530, levodopa/BAK vs levodopa:carbidopa: p = 0.340).

Administration of levodopa, levodopa/carbidopa, or levodopa/BAK drops to otherwise untreated eyes (unoccluded contralateral eyes were treated in long term experiments) did not result in any alteration in axial elongation (Wilks’ Lambda = 0.350, F(3,25) = 1.716, p = 0.074; Supplementary Table S-4) or refraction (Wilks’ Lambda = 0.362, F(3,25) = 1.650, p = 0.090; Supplementary Table S-5) relative to age-matched untreated controls. Similarly, treatment did not alter corneal thickness (ANOVA, F(3, 32) = 1.189, p = 0.339) or intraocular pressure (ANOVA, F(3, 32) = 1.355, p = 0.285; Supplementary Table S-6).

#### Levodopa treatment does not induce structural changes in retinal architecture or induce retinal cell apoptosis in chickens

Daily treatment with levodopa (15 mM; Supplementary Figure S-1C & 6E), levodopa:carbidopa (15 mM:3.75 mM; Supplementary Figure S-1 D & 6F), or levodopa:BAK (15 mM in 0.1%; Supplementary Figure S-1 E & 6G) drops over four weeks induced no observable changes in retinal structure or architecture (Supplementary Figure S-1), or observable signs of apoptosis (Supplementary Figure S-2), relative to that seen in FD or age-matched untreated control eyes (Supplementary Figures S-1 A & S-2 C).

#### Levodopa increases dopamine synthesis and release within the eye

To investigate whether levodopa administration increases dopamine synthesis and release within the eye, levels of levodopa, dopamine, and 3,4-dihydroxyphenylacetic acid (DOPAC) were measured in vitreal samples from chicks undergoing one of the following treatments: no treatment, FD, and FD with intravitreal injection of levodopa (15 mM). Samples were collected 1 hours into the light phase, which represented 1 hour of treatment. Measures of levodopa, dopamine and DOPAC levels were undertaken via liquid chromatography – mass spectrometry (LC-MS).

**Figure 5:**
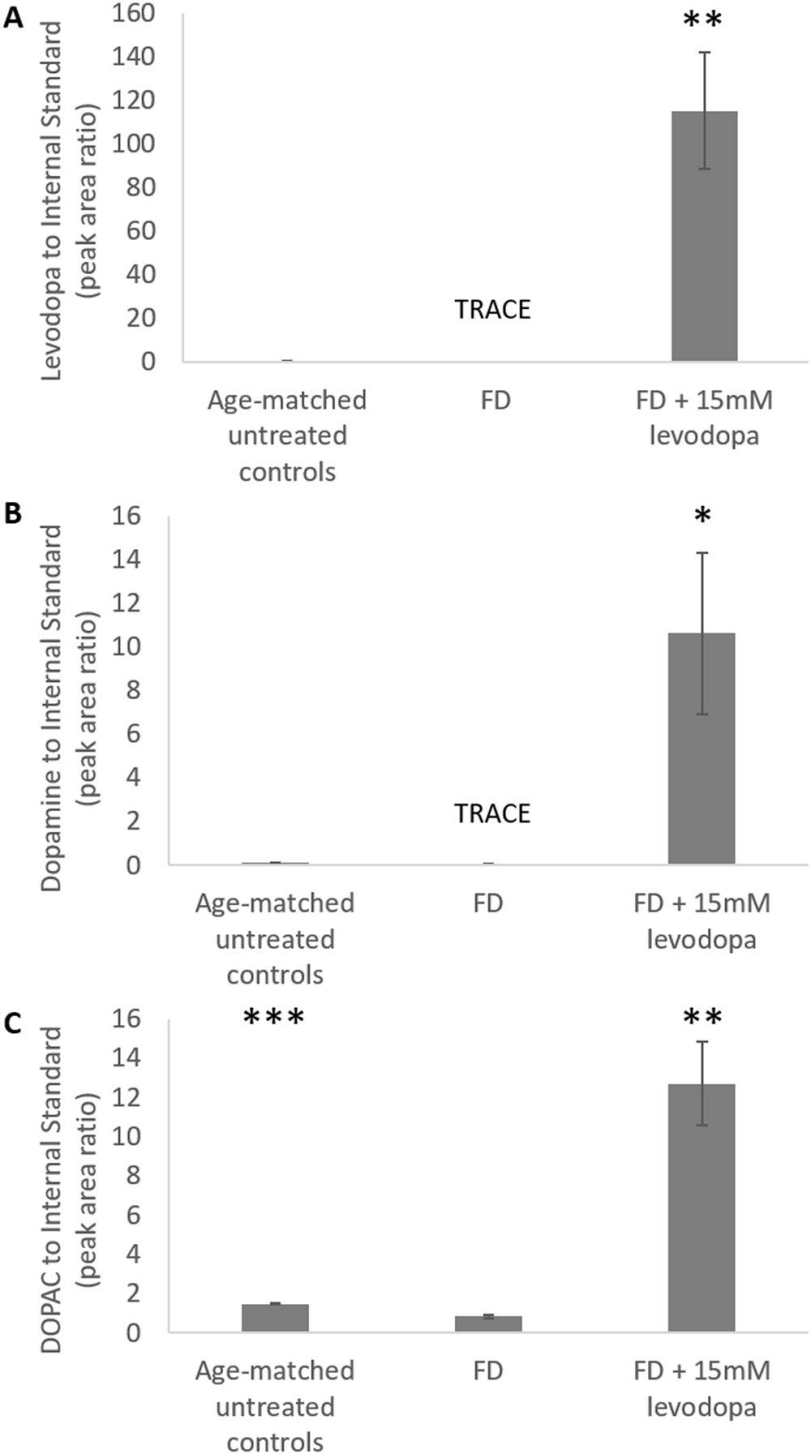
Vitreal levels of levodopa and its metabolites in form deprived (FD) and levodopa treated chicks. **(A)** Levodopa **(B)** dopamine **(C)** 3,4-dihydroxyphenylacetic acid (DOPAC). Data represents the mean ± standard error of the mean. Statistics denote difference to FD Only; *p < 0.05, **p < 0.02, ***p < 0.001. TRACE denotes when several samples were detected (S/N > 3) but not quantifiable (S/N < 10); levodopa in FD samples fell in this range in 4 out of 5 samples and dopamine in FD samples fell in this range in 2 out of 5 samples.

In FD eyes, levodopa (Fig. 5A) and dopamine (Fig. 5B) levels in the vitreous were lower, but not statistically, than that of age-matched untreated controls. The loss of statistical significance was due to the levels of both molecules falling below the quantification signal to noise cut-off (S/N 10, LOQ (dopamine: 0.28pmol/vitreous, levodopa: 1.3pmol/vitreous, DOPAC: 8.4pmol/vitreous)) in multiple samples, as such, statistics were only performed on those values falling above the signal to noise cut-off. Vitreal DOPAC however (Fig. 5C) was significantly lower in FD eyes compared to age matched untreated controls (p < 0.001). In FD eyes treated with intravitreal injections of 15 mM levodopa, vitreal levels of levodopa, dopamine, and DOPAC were substantially elevated compared to both age-matched untreated controls (p < 0.01, p < 0.05 and p < 0.01 respectively) and FD treated eyes (p < 0.01, p < 0.05 and p < 0.001 respectively).

#### Topical levodopa administration does not significantly alter levels of dopamine or its metabolites within the blood

To establish whether topical administration of levodopa increases systemic dopaminergic activity, levodopa, dopamine, DOPAC and homovanillic acid (HVA) levels were analysed by LC-MS in blood samples from untreated and levodopa treated chicks 15 minutes after administration. Blood from untreated animals was also spiked with levodopa and analysed by LC-MS immediately and after 15 minutes to simulate the maximal change expected if levodopa was systemically distributed.

**Figure 6:**
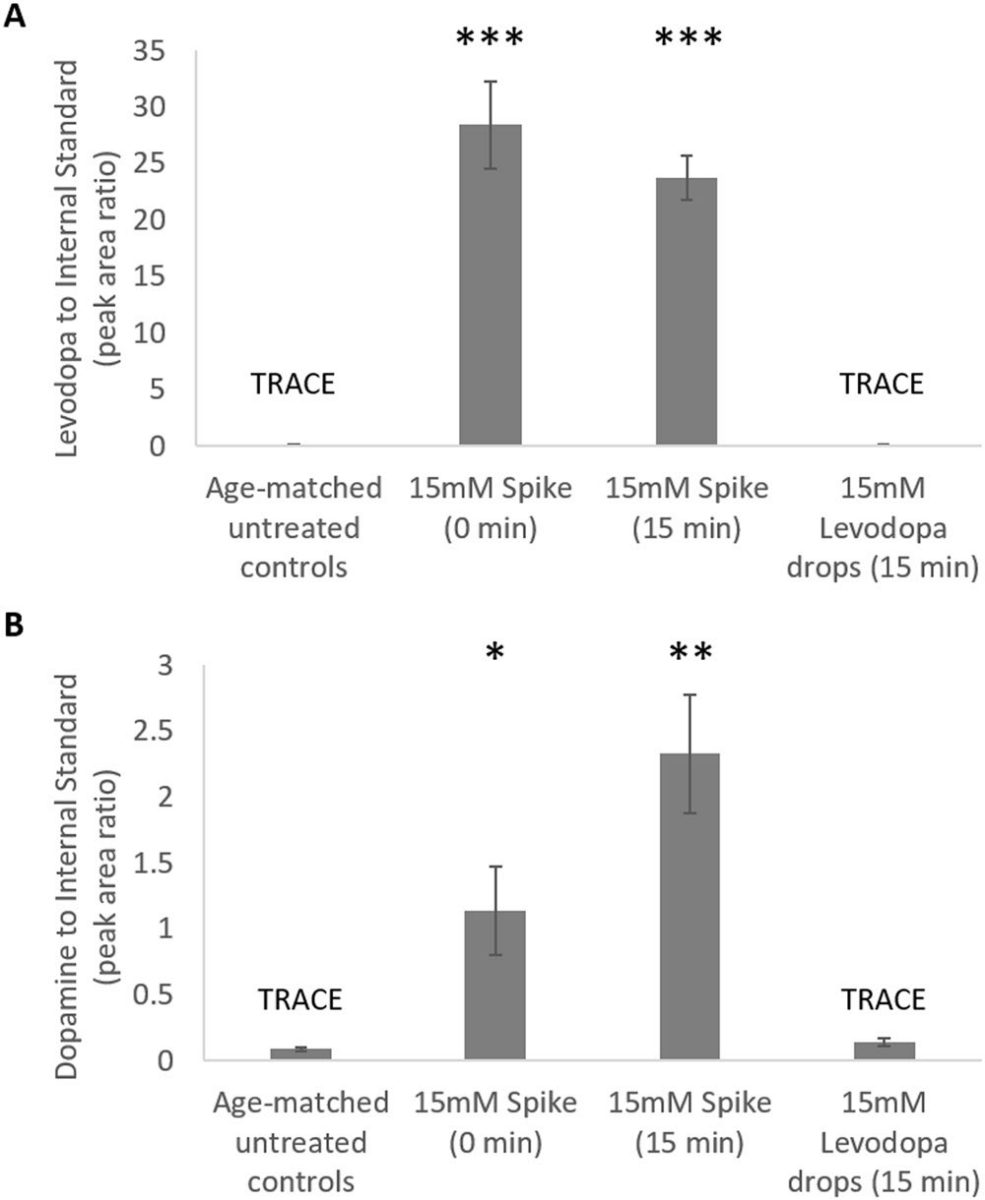
Plasma levels of levodopa and its metabolites. **(A)** Levodopa **(B)** dopamine. Data represents the mean ± standard error of the mean. Statistics denote difference to untreated controls; *p < 0.05, **p < 0.02, ***p < 0.001 (n = 9 per treatment). TRACE denotes when several samples were detected (S/N > 3) but not quantifiable (S/N < 10); levodopa in eye drops and age-matched untreated control samples fell in this range in 1 and 4 samples respectively, and dopamine in eye drops and age-matched untreated control samples fell in this range in 1 sample per group.

Direct spiking of a 15 mM levodopa solution into blood from untreated animals significantly increased levodopa (Fig. 6A) and dopamine (Fig. 6B) levels compared to age-matched untreated controls (levodopa: 0 minutes, p < 0.05; 15 minutes, p < 0.001, dopamine: 0 minutes, p < 0.05; 15 minutes, p < 0.01). Whereas topically applied levodopa (15 mM) did not result in any significant change in blood levodopa (p = 0.749) or dopamine levels (p = 0.294) from that of untreated controls. Blood from animals treated with topically applied levodopa significantly differed from both 0 (levodopa: p < 0.001, dopamine: p < 0.05) and 15 minute (levodopa: p < 0.001, dopamine: p < 0.001) spike simulations. In a number of samples, levodopa levels within untreated and drops treated animals fell below the quantification signal to noise cut-off (S/N 10, LOQ (dopamine: 5.4 ng/mL, levodopa: 170 ng/mL)), with levels well below spiked samples indicating that topically applied levodopa shows limited systemic distribution. In all groups, DOPAC and HVA were not quantifiable (S/N < 10).

#### Levodopa is of comparable effectiveness to atropine

To compare the effectiveness of levodopa in slowing ocular growth to that of atropine, the primary pharmacological treatment currently used for myopia in humans, intravitreal and topical application of atropine was tested over four days in an equivalent manner to that of levodopa (Supplementary Table S-1D & E).

**Figure 7:**
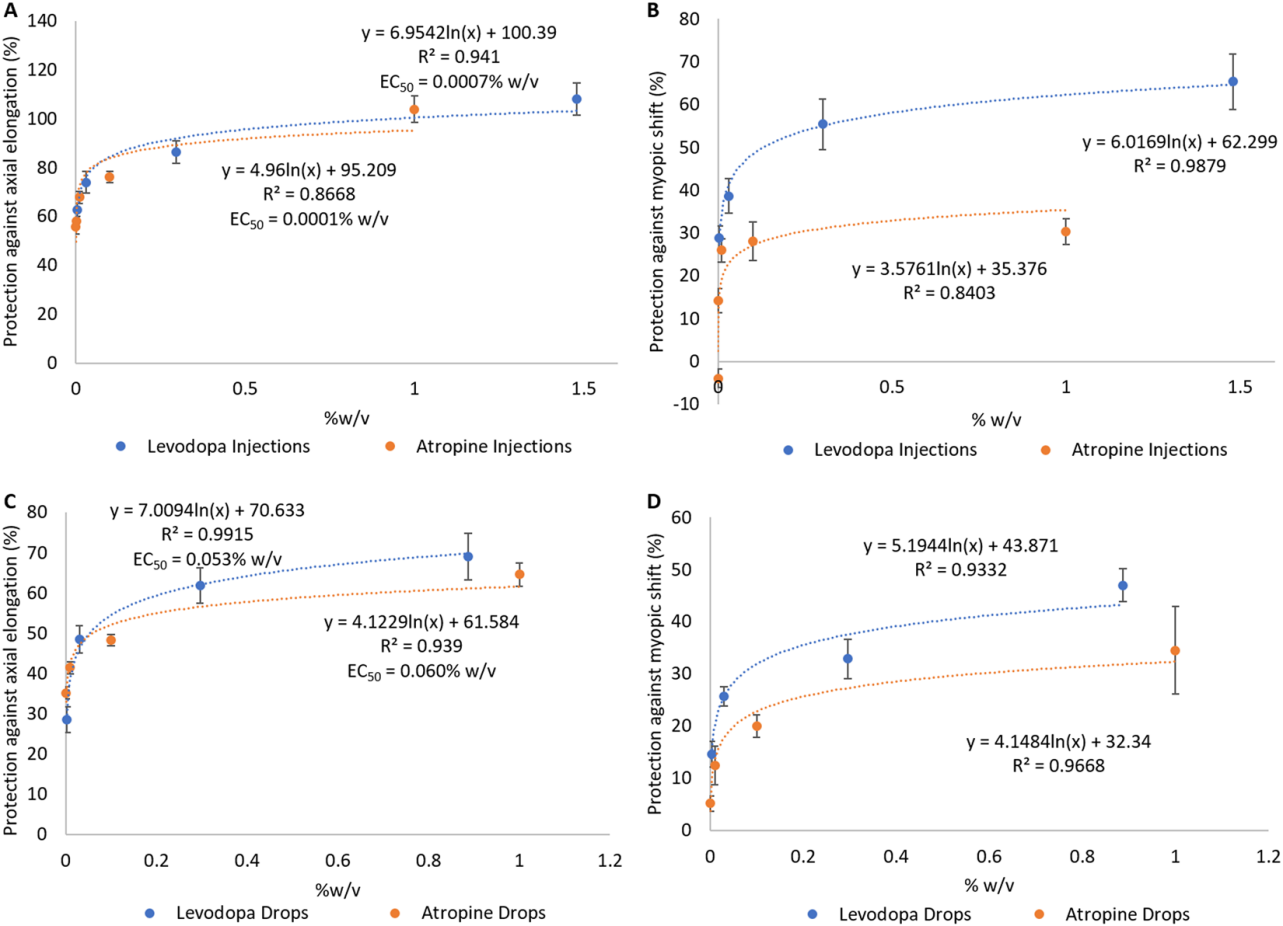
Comparison between levodopa and atropine dose response curves in chick. **(A)** Percent protection against the axial elongation and **(B)** myopic shift in refraction associated with FD in eyes treated with levodopa or atropine intravitreal injections. **(C)** Percent protection against the axial elongation and **(D)** myopic shift in refraction associated with FD in eyes treated with levodopa or atropine topical eye drops. Data represents mean ± standard error of the mean.

Intravitreal levodopa and atropine showed similar dose-dependent protection against the excessive axial growth associated with FD (levodopa vs FDM: ANOVA, F(4,68) = 15.072, p < 0.001; atropine vs FDM: ANOVA (5, 81) = 7.895, p < 0.001; atropine vs levodopa: p = 0.84; Fig. 7A; Supplementary Table S-2 and S-7). However, intravitreal levodopa showed a greater effectiveness against the myopic refractive shift associated with FD (levodopa vs FDM: ANOVA, F(4,68) = 21.654, p < 0.001; atropine vs FDM: ANOVA (5,81) = 11.535, p < 0.001; levodopa vs atropine: p < 0.001; Fig. 7B). Both compounds showed similar EC_50_ values against the axial elongation associated with FD (levodopa: 0.0007% w/v, atropine: 0.0001% w/v), with their effects best described by a logarithmic relationship (levodopa: y = 6.9542ln(x) + 100.39, atropine: y = 4.96ln(x) + 95.209).

Topical levodopa was slightly more effective than topical atropine at inhibiting axial elongation (levodopa vs FDM: ANOVA, F(4,85) = 5.494, p < 0.01; atropine vs FDM: ANOVA (4,46) = 3.632, p < 0.02; atropine vs levodopa: p < 0.05; Fig. 7C; Supplementary Table S-3 and S-7) and myopic shifts in refraction (levodopa vs FDM: ANOVA, F(4,64) = 149.165, p < 0.001; atropine vs FDM: ANOVA (4,46) = 3.922, p < 0.001; atropine vs levodopa: p < 0.05; Fig. 7D). Both compounds showed similar EC_50_ values against the axial elongation associated with FD (levodopa: 0.053% w/v, atropine: 0.060% w/v), with their protection best described by a logarithmic relationship (levodopa: y = 7.0094ln(x) + 70.633, atropine: y = 4.1229ln(x) + 61.584).

#### Long-term safety of levodopa in mice

To examine the ocular safety of levodopa in a mammalian model (mouse), complementing the results observed in chicks above, levodopa and levodopa/carbidopa eye drops were administered to otherwise untreated C57BL/6 J mice (Supplementary Table S-1E) over a period of 9 months.

No abnormalities were detected in daily health score analysis of treated or untreated mice over 9 months of treatment whilst base histology and apoptosis analysis, like chicks, showed no signs of retinal degradation or toxicity (data not shown).

Further, normal refractive development (age-matched untreated control males: 6.37 ± 0.17D; females: 4.82 ± 0.46D) was not altered by chronic administration of 15 mM levodopa (males: 6.55 ± 0.41D; females: 5.24 ± 0.41D) or 15 mM levodopa with 3.75 mM carbidopa drops (males: 6.78 ± 0.16D; females: 5.33 ± 0.60D) following nine months of treatment (ANOVA F(2,27) = 1.471, p = 0.239).

**Figure 8:**
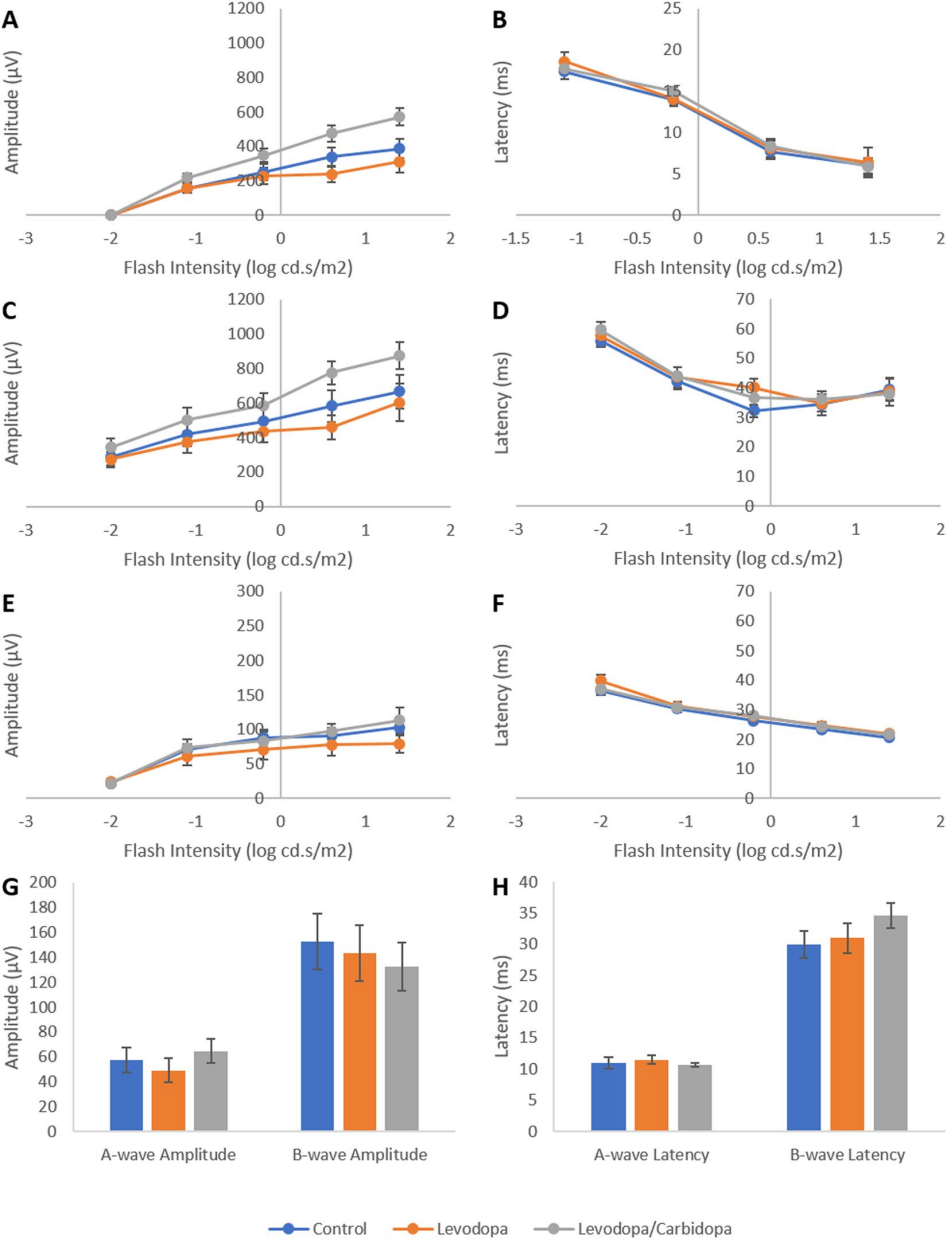
Effects of chronic (9-months) topical application of levodopa or levodopa/carbidopa on mouse electroretinogram responses. **(A)** A-wave amplitude; **(B)** A-wave latency; **(C)** B-wave amplitude; **(D)** B-wave latency; **(E)** Oscillatory potential 2 amplitude; **(F)** Oscillatory potential 2 latency. **(G)** Amplitude of responses in light adapted animals. **(G)** Latency of responses in light adapted animals. Data represents mean ± standard error of the mean.

Electroretinogram responses are not altered by topical application of levodopa: Chronic application of levodopa (15 mM) or levodopa/carbidopa (15 mM/3.75 mM) over a 9-month period did not induce any permanent changes in recorded electroretinogram (ERG) waveforms (Supplementary Figure S-3) or waveform parameters (Fig. 8) following a 5 day washout period. Specifically, no differences occurred between treated and untreated animals in A-wave amplitude (Wilks’ Lambda = 0.763, F(2,25) = 1.314, p = 0.086), or latency (Wilks’ Lambda = 0.788, F(2,25) = 1.468, p = 0.181) or, B-wave amplitude (Wilks’ Lambda = 0.675, F(2,25) = 1.869, p = 0.061) or latency (Wilks’ Lambda = 0.690, F(2,25) = 1.752, p = 0.082), or the amplitude (Wilks’ Lambda = 0.748, F(2,25) = 1.219, p = 0.292) or latency (Wilks’ Lambda = 0.749, F(2,25) = 1.211, p = 0.297) of oscillatory potential 2. Furthermore, no significant differences arose in light adapted responses (Wilks’ Lambda = 0.498, F(2,25) = 1.458, p = 0.217).

Similarly, levodopa administration had no notable effect on ERG waveforms (Supplementary Figure S-4) or waveform parameters (Supplementary Figure S-5) in recordings performed 2 hours following topical levodopa administration to previously naïve mice.

## Discussion

Intravitreal and topical application of levodopa slowed ocular growth and significantly inhibited the development of experimental myopia (FDM) in a dose-dependent manner. The EC_50_ values for both intravitreal injection and tropical application of levodopa were similar to those of atropine, the gold standard for pharmacological treatment of myopia in humans. Intravitreal injection of levodopa slowed the excessive ocular growth associated with FDM to a greater extent than that of topical application. However, the addition of carbidopa enhanced the effects of topically applied levodopa, similar to that seen during its systemic use for the treatment of neurological disorders, by presumably inhibiting its premature break-down by the enzyme aromatic l-amino acid decarboxylase (AAAD)^29^, and thus lowering the dose of levodopa required to achieve protection. Interestingly, the protective effects of systemic levodopa against the development of experimental myopia in guinea pigs have been reported to be inhibited by injection of carbidopa into the peribular space^30^, contrary to what would be expected from the existing literature surrounding the coadministration of levodopa and carbidopa.

The effectiveness of topically applied levodopa and levodopa:carbidopa was maintained over a period of 4 weeks (chronic treatment), which represents approximately one third of the ocular maturation period of chicks^31,32^ and the critical window in which eye growth is most susceptible to visual manipulation in this species^32^. As seen during acute treatment, levodopa’s effectiveness was significantly enhanced by co-administration with carbidopa. In contrast, the addition of the preservative and corneal penetrating agent BAK did not enhance the protective effects seen relative to levodopa alone.

Chronic application of levodopa, or levodopa:carbidopa, as a topical solution did not induce changes in those measures of ocular health examined in chicks (histology and cell survival, intraocular pressure, or normal ocular development (ocular biometry and refraction)) and mice (ERG recordings, histology and cell survival, and normal refractive development). The lack of alterations in ERG recordings in mice mirrors work in Parkinsonian patients, in which levodopa treatment does not alter normal ERG responses^33^ and has been shown to retrieve waveform parameters in subjects with abnormal ERG responses associated with depressed dopaminergic activity in Parkinson’s disease^34–36^. Furthermore, no alterations in normal ERG responses are observed following high doses of levodopa (200 mg)^37,38^. In accordance with a lack of change in the electrical activity of the retina, levodopa did not induce alterations in retinal architecture or produce signs of cell stress/death (apoptosis). In agreement with these findings, chronic levodopa administration has not been associated with ocular complications in patient populations^33–38^. This is consistent with the literature surrounding levodopa and cell health, as although levodopa has been reported to cause apoptosis in cultured neural tissue^39^, when administered *in vivo*, little toxicity has been demonstrated in neurologically normal^40,41^ or neurologically vulnerable^42–44^ animals (for review see^45^). Furthermore, clinical studies have indicated that chronic levodopa treatment is non-toxic to nigrostriatal dopaminergic neurons in humans^46–48^. Importantly, we report that levodopa does not appear to alter normal ocular development, and instead only becomes effective when administered to eyes in which the retinal dopaminergic system is dysregulated by myopia induction.

Together, the findings of this study suggest that levodopa may be a potential therapeutic treatment for human myopia by acting to restore dopaminergic function. Based on recent clinical findings, topical application of levodopa may also have potential for the treatment of other visual disorders in which dysregulation of dopaminergic system has been implicated and where systemic levodopa treatment has been shown to be beneficial, including; amblyopia^49^, diabetic retinopathy^50^ and age-related macular degeneration (AMD)^51^. Levodopa has been in clinical use for over five decades and is the primary treatment for neurological disorders, such as Parkinson’s disease, involving the dysregulation of the dopaminergic system (for review see^28^). Systemic administration of levodopa, as undertaken for Parkinson’s disease, is not appropriate for the treatment of myopia as it would lead to changes in dopaminergic activity not only within the eye, but within the brain and the rest of the body in an otherwise healthy developing child. Topical application of levodopa would maximise the amount of levodopa reaching the retina while minimising systemic distribution, lowering the dose required to achieve protection relative to systemic administration. Accordingly, levodopa administered via eye drops did not significantly alter dopaminergic activity within the blood, particularly when compared to the elevated levels observed in spiked samples. This suggests that, when myopia inhibiting doses are applied to the eye, limited systemic distribution of levodopa occurs. Therefore, this approach minimises the likelihood of those side effects most commonly reported with long-term levodopa treatment which can be exacerbated at higher concentrations (such as motor fluctuations and dyskinesias^28^). There is currently no clinical data available on the use of topical levodopa, in ophthalmic preparations or elsewhere, so it is difficult to evaluate the degree to which levodopa eye drops may induce side effects arising from systemic distribution. Studies on systemic administration of levodopa in children with amblyopia have implicated side effects such as headaches, nausea, fatigue, mood changes, nightmares, emesis, dizziness, dry mouth and decreased appetite^52^. Elevated dopamine levels in the brain have been linked to attention deficit disorders. However, whether these disorders are driven by increased dopamine release, or whether elevated dopamine levels are a consequence of the pharmacological treatment of these disorders is still under debate^53^. Levodopa has also been associated with cardiovascular effects^54,55^ and schizophrenia^56,57^ in a subset of individuals, although such an association requires further investigation. Our current data indicates that topical levodopa application did not lead to any notable side effects or adverse effects, however, care must always be taken when modulating dopaminergic activity.

The dose-response curve for atropine eye drops in this study correlated remarkably well with results observed in its clinical use (for review see^58^). Specifically, similar to the EC_50_ we report here (0.06% w/v), 0.05% w/v atropine has been shown to slow myopia progression in children by roughly 60%^59^. This suggests that the animal pharmacological EC_50_ data correlates well with observed clinical outcomes. Therefore, as the dose curves for topically applied atropine and levodopa are markedly similar, this would suggest that the EC_50_ for levodopa may translate into clinical outcomes with a similar consistency to that of atropine, however this does not account for differences in the mechanism of action of levodopa and atropine.

While atropine is a well-validated tool for controlling the progression of myopia in humans, its side-effects at high doses^60^, the existence of a rebound effect after cessation of treatment^61^, uncertainties about its mode of action^62^, and the reported existence of non-responders, suggest that an alternative pharmacological treatment may be useful. These findings suggest that topical application of the naturally occurring compound levodopa, which has a well-documented history of use in humans, may be a viable candidate for future clinical treatment of myopia.

As excessive axial elongation is the root cause of pathological outcomes arising from myopia, and therefore the primary clinical target, more attention was afforded the ability of levodopa to attenuate axial elongation. In this study, as is commonly exhibited in animal models of myopia^63–72^, axial length and refraction data demonstrate the same direction of change, despite differences in overall effect size, with no alterations were observed in other ocular components (corneal curvature, corneal thickness, anterior chamber depth, and lens thickness). Chicks were refracted in darkened rooms, however, cycloplegia was not induced as the required use of nicotinic antagonists, which are known to themselves inhibit experimental myopia^64^, could lead to a misrepresentation of the effectiveness of levodopa.

### Conclusions

In chickens, manipulation of the dopaminergic system by intravitreal or topical administration of levodopa slows ocular growth and thereby inhibits the development of experimental myopia in a dose-dependent manner. Levodopa shows a similar potency to that of atropine, currently the primary pharmacological treatment for myopia in humans. The addition of carbidopa significantly enhances the potency of topical levodopa, allowing its growth inhibiting effects to be maintained at much lower doses. Further, topically applied levodopa and levodopa/carbidopa remains effective over chronic treatment periods. Importantly, topical application of levodopa or levodopa:carbidopa did not alter visual function and ocular health in those parameters tested in chicks or mice. These findings indicate that levodopa may have potential as a therapeutic intervention for human myopia.

## Methods

### Overview

The effects of levodopa upon ocular growth were initially examined by testing the dose-dependent effects of intravitreal and topical application of levodopa against the development of form-deprivation myopia (FDM) in chicks over a period of 4 days. Within this 4 day treatment period, the effects of intravitreal and topical atropine, as well as ways to increase the effectiveness of topical levodopa through the addition of carbidopa or DMSO, were also studied in the chick form-deprivation model.

To complement the above work, the effects of topical levodopa and levodopa/carbidopa against the development of FDM were examined in chicks over a chronic, 4 week period, which was combined with an analysis of ocular safety focusing on retinal health, refractive development, axial elongation, and interocular pressure.

To show that levodopa treatment altered dopamine levels within the eye, but not systemically, catecholamine levels were quantified in the vitreous body and blood samples. This analysis was undertaken in form-deprived chicks following a single treatment of levodopa. Chicks were not analysed after four days or four weeks in this initial experiment to remove the compounding factor of potential adaptation with time.

With a focus on possible clinical use in humans, ocular safety findings from chicks were translated to a mammalian model (C56BL/6 J mice). Topical eye drops were administered to otherwise untreated mice for a period of 9 months to complement the avian data and examine the effects of chronic treatment on visual function, retinal health, and normal refractive development this mammalian model.

### Animal housing

Day-old male White-Leghorn chickens were obtained from Barter & Sons Hatchery (Horsley Park, NSW, Australia). Chicks were kept in temperature-controlled rooms and were given five days to adjust to their surroundings before the experiments commenced. Chicks had access to unlimited amounts of food and water and were kept under normal laboratory lighting (500 lux, fluorescent lights) on a 12:12 hour light:dark cycle with lights on at 9:30am and off at 9:30 pm.

Mice (C56BL/6 J) were obtained from the University of Canberra breeding colony. Mice were kept five to a cage (males and females caged separately) in individually ventilated caging, with experiments commencing at 8 weeks of age. Mice were housed at 23 °C on a 12:12 hour light:dark cycle (8am:8 pm; 500lux fluorescent lights) and had access to unlimited amounts of food and water.

Experimental procedures were approved by the University of Canberra Animal Ethics Committee under the ACT Animal Welfare Act 1992 (project number CEAE 16–05) and conformed to the ARVO Statement for the Use of Animals in Ophthalmic and Vision Research.

### Experimental methods – short-term (4 day) effectiveness of levodopa in chicks

#### Standard experimental structure

Myopia was induced by placing a translucent diffuser (FDM) over the treated (left) eye as previously described to induce form-deprivation myopia (FDM)^25^. Specifically, Velcro mounts were fitted around the left eye on the day prior to the commencement of treatment. On the following day, a diffuser fitted to a matching Velcro ring was mounted over the eye. The right eyes remained untreated to serve as an internal contralateral control.

For drug administration, chicks were given a once daily 10 μL intravitreal injection (9:30am), or two 40 μL topical eye drops twice daily (9:30am and 2 pm), of their respective drug solution (levodopa, levodopa/caribodpa, levodopa/DMSO or atropine) to their diffuser-treated (left) eye for a period of four days. For intravitreal administration, chicks were anaesthetised under light isoflurane (5% in 1 L of medical grade oxygen per minute, Veterinary Companies of Australia, Kings Park, NSW, Australia) using a vaporiser gas system (Stinger Research Anaesthetic Gas Machine (2848), Advanced Anaesthesia Specialists, Payson, Arizona, USA).

For all preparations, levodopa (Sigma Aldrich, D9628) was dissolved fresh in a solution containing 0.1% w/v ascorbic acid in 1x phosphate-buffered saline (PBS). Immediately prior to administration, the pH of the levodopa solution was adjusted to 5.5. For treatments containing carbidopa (Sigma Aldrich, C1335), levodopa and carbidopa were combined at a 4:1 ratio and dissolved as per outlined above for levodopa alone. For treatment with dimethyl sulfoxide (DMSO), levodopa was dissolved as above with the addition of 10% v/v DMSO (Sigma Aldrich, D8418). Atropine solutions comprised of atropine monosulfate (Abcam, A10236) dissolved in 1xPBS (pH of 6).

Refraction and ultrasonography measurements, to assess myopia development, were undertaken the day prior to the commencement of treatment and on the final day of the experimental period as previously described^25^. Refraction was measured using automated infrared photoretinoscopy (system provided courtesy of Professor Frank Schaeffel, University of Tuebingen, Germany). Measurements were taken for both treated (left) and contralateral control (right) eyes, with refractive values representing the mean spherical equivalent of 10 measurements per eye. For axis alignment, the Purkinje image was centered within the pupil to obtain the correct refractive axis, with illumination levels within the room held at less than 5 lux to avoid light reflections in the pupil arising from aberrant sources. Axial length was measured, on chicks anesthetised as above, using A-scan ultrasonography (Biometer AL-100; Tomey Corporation, Nagoya, Japan). For short-term experiments, ocular parameters for the drug treated eyes (Supplementary Table S-1) were compared against diffuser-treated animals without levodopa (FDM Only, n = 26), age-matched untreated control animals (n = 17), and their contralateral control eyes.

#### Levodopa dose-response curve (intravitreal injections)

To investigate whether levodopa, when introduced directly to the retina (Supplementary Table S-1A), can prevent FDM in chickens, animals were divided between the following groups:

1. Fitted with a translucent diffuser – daily intravitreal injection of levodopa at one of the following concentrations: 0.15 mM (n = 8), 1.5 mM (n = 15), 15 mM (n = 15), 75 mM (n = 8);
2. Fitted with a translucent diffuser – daily intravitreal injection of the vehicle solution (0.1% w/v ascorbic acid in 1xPBS, pH 5.5; n = 10);
3. No translucent diffuser fitted – daily intravitreal injection of levodopa (15 mM, n = 10).

#### Levodopa dose-response curve (topical drops)

To establish whether a protective effect against the development of FDM in chicks persists when levodopa is administered as a topical solution to the eye (Supplementary Table S-1B), and whether this delivery of levodopa can be enhanced by the addition of carbidopa (to prevent premature metabolism) or DMSO (to enhance corneal penetration), chicks were divided between the following groups:

1. Fitted with a translucent diffuser – topical application of levodopa at one of the following concentrations: 0.15 mM (n = 18), 1.5 mM (n = 18), 15 mM (n = 14), 45 mM (n = 9);
2. Fitted with a translucent diffuser – topical administration of 15 mM levodopa with 3.75 mM carbidopa (n = 14), or 15 mM levodopa in 10% DMSO (n = 9);
3. Fitted with a translucent diffuser – topical application of the vehicle solution alone (0.1% w/v ascorbic acid in 1xPBS, pH 5.5; n = 11);
4. No translucent diffuser fitted – topical application of levodopa (15 mM, n = 15).

Due to solubility limits, levodopa at a concentration of 75 mM or higher will not remain in solution for longer than 10 minutes at a pH 5.5. Therefore, a 45 mM solution, which sat at the upper solubility limit of levodopa at pH 5.5, was the highest dose tested for topical eye drops.

For all further analyses of levodopa, a concentration of 15 mM was used. The higher dose of 45 mM was not used for further analyses as although it shows a stronger treatment effect, it sits at the solubility limit of this compound.

Following 4 days of topical treatment with a 15 mM levodopa solution into form-deprived eyes, corneal thickness (DGH Pachmate 2 pachymeter, DGH Technology Inc., USA) and corneal curvature were measured to determine any effects on the refractive surface. Corneal curvature (measured as the radius of curvature) was examined following the procedure outlined in Troilo & Wallman^73^ using a keratometer (Topcon OM-4) fitted with a + 8D lens to adapt the system to the highly curved chick cornea, and calibrated by measuring curvatures of chrome balls of known diameters (2–8 mm).

#### Effectiveness of levodopa relative to atropine (intravitreal and topical application)

To examine the effectiveness of levodopa when compared to that of atropine, the most widely used pharmacological treatment for myopia in humans, a dose-response analysis of atropine was also undertaken (Supplementary Table S-1C,D). For this, chicks were divided between the following groups:

1. Fitted with a translucent diffuser – intravitreal injection of atropine at one of the following concentrations: 0.0015 mM (n = 12), 0.015 mM (n = 12), 0.15 mM (n = 12), 1.5 mM (n = 12), 15 mM (n = 12);
2. Fitted with a translucent diffuser – topical application of atropine at one of the following concentrations: 0.015 mM (n = 6), 0.15 mM (n = 6), 1.5 mM (n = 6), 15 mM (n = 6).

### Experimental methods – long-term (4 week) effectiveness and safety of levodopa in chicks

To establish the effectiveness of levodopa over a more chronic treatment period, myopia was induced, and topical levodopa administered to chicks as outlined for 4 day treatments, for a period of 4 weeks. Refraction and ultrasonography measurements were undertaken prior to the commencement of the experiment and at the end of each proceeding week as outlined for short-term experiments. In addition, corneal thickness (measured as above in 4 day experiments) and intraocular pressure (Icare TA01i tonometer, Icare, Finland) of chicks were also measured alongside the final biometric measurements after 4 weeks of treatment. Ocular parameters for the drug treated eyes (Supplementary Table S-1B) were compared against diffuser-treated animals without levodopa (FDM Only, n = 18) and age-matched untreated control animals (n = 18).

Over this 4 week period, chicks were administered eye drops to both eyes, with the left eye fitted with a translucent diffuser and the right eye receiving no optical treatment. Levodopa treatment animals were divided into 3 groups (Supplementary Table S-1B):

1. Fitted with a translucent diffuser – topical application of 15 mM levodopa (n = 18);
2. Fitted with a translucent diffuser – topical application of 15 mM levodopa/3.75 mM carbidopa (n = 12);
3. Fitted with a translucent diffuser – topical application of 15 mM levodopa in 0.1% w/v benzalkonium chloride (BAK, n = 6).

Levodopa and levodopa/carbidopa solutions were prepared as outlined for 4 day treatments. For treatment with BAK, levodopa was dissolved as above with the addition of 0.1% w/v BAK (Sigma Aldrich, 12060). BAK was used in place of DMSO due to its higher frequency of use in topical ophthalmic preparations as an antimicrobial and corneal penetrating agent^74^. Following final measurements, chicks were sacrificed and tissue prepared for histological analysis following procedures outlined by Karouta & Ashby^25^ (see Supplementary Methods for full details). Retinal sections were treated with toluidine blue to examine retinal architecture or underwent TUNEL analysis to determine whether the retina was undergoing apoptosis.

### The effects of levodopa treatment on catecholamine levels in previously naïve chicks

#### Ocular dopamine levels

To show that levodopa treatment altered dopamine levels within the eye, catecholamine levels were quantified in the vitreous body samples. This analysis was undertaken in form-deprived chicks following a single treatment of levodopa. Chicks were not analysed after four days or four weeks in this initial experiment to remove the compounding factor of potential adaptation with time.

Vitreous samples were measured using high performance liquid chromatography tandem mass spectrometry (LC-MS-MS) following a protocol adapted from Perez-Fernandez *et al*.^75^ (see Supplementary Methods for full details). Samples were collected 1 hour after drug treatments and the fitment of diffusers to previously naïve chicks. Treatment commenced at lights on (9:30am), with chicks broken into the following groups:

1. Fitted with a translucent diffuser – intravitreal injection of levodopa (15 mM, n = 5);
2. Fitted with a translucent diffuser – no intravitreal or topical application of the drug (myopia control group (FD Only), n = 5);
3. Age-matched untreated control animals (n = 5).

#### Systemic (blood) dopamine levels

To examine if levodopa is systemically distributed following topical application, and whether topical levodopa application alters dopamine levels systemically, blood samples from chicks treated with 15 mM drops were measured using an LC-MS-MS following a protocol adapted from Perez-Fernandez *et al*.^75^ (see Supplementary Methods for full details). Samples were collected 15 minutes after drug treatment to examine the maximum amount of systemically distributed levodopa, with topical application occurring at lights on (9:30am). Chicks were divided into the following two groups:

1. Topical application of levodopa (15 mM, n = 9);
2. Age-matched untreated control animals (n = 9);

In a concurrent experiment, aliquots of blood taken from the above age-matched untreated animals were spiked with a 15 mM levodopa solution. The volume of the spike was adjusted relative to the volume of blood taken. That is, at this age the chicken contains 8 mL of blood^76^. From this 8 mL total blood volume, 1 mL aliquots were taken, which represents 12.5% of the total blood volume of the animal. Therefore, 12.5% of the topical volume, which is normally 0.08 mL, was added to the blood. Spiked blood samples were then analysed at two timepoints (time zero (n = 9) and following 15 minutes incubation at room temperature (n = 9)). This process simulated the maximum levodopa that should be detectable systemically if the entirety of the topical drops were absorbed into the circulation.

### Mammalian analysis of safety (mouse)

#### Chronic levodopa treatment

To investigate the safety of levodopa administration in a mammalian model, C57BL/6 J mice were divided into 3 treatment groups (Supplementary Table S-1E) and chronically treated (9 months):

1. Age-matched untreated controls (5 males, 5 females);
2. Topical levodopa treatment (15 mM) to otherwise untreated eyes (5 males, 5 females);
3. Topical levodopa/carbidopa treatment (4:1, 15 mM:3.75 mM respectively) to otherwise untreated eyes (5 males, 5 females).

Commencing at 8 weeks of age, mice were administered one 10 µL drop of their respective drug solution (Supplementary Table S-1E) to each eye once daily at 11am or left untreated, for a period of 9 months. Each day, a health score analysis was undertaken that examined weight, fur condition, activity, respiratory rate, gait, interactions with casemates, temperament, faeces, posture, and external condition of the eye.

Refraction was measured at the completion of the 9-month period using infrared photorefraction as outlined above for chickens, with the 75 mm F/1.4 C-type lens with a 5 mm extension tube replaced with a 50 mm F/1.4 C-type lens with a 10 mm extension tube to account for the smaller eye of the mouse. Following a further 5 days of no topical treatment (washout period), ERG measurements (see Supplementary Methods for full details) were undertaken before the mice were sacrificed by an overdose of isoflurane anaesthetic, with eyes immediately enucleated for base histology and TUNEL analysis (see Supplementary Methods for full details).

In addition to examining ERG responses following chronic treatment (9 months) with levodopa, we also examined whether retinal function is immediately altered following daily levodopa application. To do this, previously untreated mice of an equivalent age (9 months) to those treated above were administered one drop of 15 mM levodopa to each eye (n = 3 males, n = 2 females). Two hours after drops were given, during which time the animals remained in the dark, ERG recordings were performed and compared to those of age-matched untreated control animals (n = 3 males, n = 2 females).

### Statistical analysis

All values reported represent the mean ± the standard error of the mean. Before analysing the effect of treatment, all data were first tested for normality and homogeneity of variance (Shapiro-Wilk test). Following this, the effect of short-term treatment was analysed via a one-way univariate analysis of variance (ANOVA) followed by a student’s unpaired *t*-test, with Bonferroni correction for multiple testing, for analysis of specific between group effects. ERG responses and the effects of long-term treatment were analysed using a repeat measures multivariate analysis of variance (MANOVA) followed by a student’s unpaired *t*-test, with Bonferroni correction for multiple testing, for analysis of specific between group effects. All analyses were undertaken in IBM SPSS Statistics package 23 with a statistical cut-off of 0.05.

## Supplementary information

Supplementary Information

## Data Availability

All data generated or analysed during this study are included in this published article (and its Supplementary Information files).
