# Supplementary material for: Effectiveness and safety of topical levodopa in a chick model of myopia
Source: Sci Rep. 2019 Dec 4;9:18345. doi: 10.1038/s41598-019-54789-5 (PMC6892936; doi:10.1038/s41598-019-54789-5)
Supplement: Supplementary file 1 — Supplementary Information [file 41598_2019_54789_MOESM1_ESM.docx]

Effectiveness and safety of topical levodopa in a chick model of myopia

Supplementary Material

Kate Thomson, Cindy Karouta, Ian Morgan, Tamsin Kelly and Regan Ashby

# Supplementary Methods

## Histology

To investigate the effect of levodopa on retinal structure, architecture and cell health in chicks, base histology with toluidine blue staining and a terminal deoxynucleotidyl transferase dUTP nick end labelling (TUNEL) assay were performed. Chicks were sacrificed by an overdose of isoflurane anaesthetic followed by decapitation, with the eyes immediately enucleated and hemisected. The posterior eye cup was fixed in freshly prepared 4% paraformaldehyde (Sigma, P6148) in 1x phosphate buffered saline (1xPBS, pH 7.4) for 4 hours at 4˚C. After fixation, the eye cups were washed in three changes of 30% sucrose solution (Sigma, S9378) and cryoprotected overnight in 30% sucrose at 4˚C. Eye cups were then embedded in OCT mounting medium at -20˚C (Tissue Tek), before 12-15 µm cryo-sections were cut on a Leica CM1860 cryostat and mounted on superfrost microscope slides (Menzel-Glaser) before being dried for 1 hour at room temperature. Retinal sections were taken from the area centralis and at 100 and 300μm into the eye from a superior nasal orientation and stored at -20˚C until use.

For analysis of general histology, retinal tissue was stained with toluidine blue. For staining, sections were immersed in a 1% w/v toluidine blue solution (Sigma Aldrich, 89640; dissolved in distilled water) for 1.5 minutes prior to rinsing under running distilled water for 2 minutes. Sections were then mounted in glycerol before cover slipping. Sections were viewed on a Motic BA410 light microscope at 20X or 40X magnification and captured by a Moticam 10-megapixel camera in conjunction with the Motic Live Imaging Module.

To examine the retina for signs of apoptosis, a TUNEL assay was performed. TUNEL staining was undertaken using the Roche *In Situ* Cell Death Detection Kit, AP (Sigma, 11684795910) following a protocol adapted from the work of Denton & Kumar (Cold Spring Harbor Protocols, 2015) and the manufacturer’s instructions. In short, sections were prepared by washing in three changes of 1xPBS in 0.1% v/v Triton X-100 (1xPBST), permeabilising in 0.1% w/v sodium citrate on ice for 5 minutes, followed by washing in 3 more changes of 1xPBST. Sections were then incubated in TUNEL Reaction Mixture according to the manufacturer’s instructions (negative controls were incubated in Labelling Mixture only, whilst positive controls were incubated in DNase 1 (100 U/mL) for 30 minutes prior to TUNEL labelling to induce DNA fragmentation like that observed in apoptosis). Following TUNEL labelling, sections were washed in the dark in 3 changes of 1xPBS, followed by visualisation using a Leica DMIL fluorescent microscope at 20X magnification with images captured by a Leica DFC425 camera using the Leica Application Suite version 4.8.

## LC-MS-MS

**Vitreous Sample Preparation**

One hour after treatment chicks were sacrificed using an overdose of isoflurane. Eyes were rapidly enucleated and hemisected, so as to obtain the vitreous body, which were stored at -80°C until analysis.

In short, samples were homogenised for 1 minute in 90 μL of 0.5mM ascorbic acid in 1% (v/v) formic acid in MilliQ water and 10 μL of an internal standard mix. The internal standard mix consisted of 1 μg/mL dopamine-d_4_ HCl (as free base, Cerilliant D-072), 12 μg/mL DOPAC-d_5_ (Sigma, 778206), and 6 μg/mL levodopa-d_3_ (as free base, Sigma, 333786) in 0.5 mM ascorbic acid in 1 % (v/v) formic acid in MilliQ water. Samples were then sonicated in ice-cold water for 5 minutes and centrifuged at 14000 rpm (20800g) for 45 minutes at 4 °C; the supernatant (80 μL) was then analysed by LC-MS-MS.

**Blood Sample Preparation**

Samples were centrifuged at 14000 rpm (20800g) for 45 minutes at 4 °C before 100 μL of an internal standard mix was added. The internal standard mix consisted of 1 μg/mL dopamine-d_4_ HCl (as free base, Cerilliant D-072), 12 μg/mL DOPAC-d_5_ (Sigma, 778206), 6 μg/mL levodopa-d_3_ (as free base, Sigma, 333786) and 6 μg/mL HVA-d_5_ (Cerilliant, H-092) in 0.5 mM ascorbic acid in 1 % (v/v) formic acid in MilliQ water. Samples were then sonicated in ice-cold water for 5 minutes and centrifuged at 14000 rpm (20800g) for 45 minutes at 4 °C; the supernatant (80 μL) was then filtered through 4mm 45μm nylon syringe filters (Thermo fisher) prior to LC-MS-MS analysis.

**General Conditions**

Samples were analysed using an Agilent 1260 Infinity HPLC interfaced with an Agilent 6410 triple quadrupole mass spectrometer, equipped with an ElectroSpray ionisation (ESI) source. All data was acquired and quantified using MassHunter software (Version B 04.01). The separation was achieved on an Agilent InfinityLab Poroshell 120 EC-C18 analytical column (dimensions 2.7 μm, 3.0 x 50 mm; Agilent, 699975-302), fitted with a frit and a corresponding guard column (dimensions 2.7 μm, 3.0 x 5 mm; Agilent, 823750-911). A gradient elution with a binary mobile phase system of (A) 0.1 % v/vformic acid in MilliQ water and (B) 0.1 % v/v formic acid in LC-MS grade methanol was performed, with a column temperature of 40 ^o^C and a 0.2 mL/min flow rate. The gradient profile was 5 % B held for 2 minutes, increasing to 100 % B over 6 minutes, and then held for 5 minutes, prior to re-equilibration at 5 % B for 12 minutes (resulting in a 25 minute analysis time, divided into the 2 time segments based on MS ionisation mode). The autosampler was maintained at 4 ^o^C and an injection volume of 20 μL was used. After analysis, the column was back-flushed overnight with 100 % LC-MS grade MeOH at 0.35 mL/min.

**MRM Parameters for Vitreous Samples**

Optimised multiple reaction monitoring (MRM) parameters are summarised in Table S-8A. The corresponding molecular ion and up to three most predominant fragment ions were utilised for each analyte; DOPAC and its deuterated standard had one MRM transition monitored respectively due to the lack of any additional sufficiently intense fragment ions. Additional MS parameters were as follows: gas temperature and flow rate 340 ^o^C and 8.5 L/min, nebuliser 25 psi, capillary voltage 3000 V (positive) and 2000 V (negative), cell acceleration voltage 7 V. Both quadrupoles were operated in unit resolution.

The limit of quantification (LOQ) of each analyte in the vitreous matrix was estimated based on a signal to noise ratio of 10:1 for the deuterated quantifier MRM transition and an injection volume of 20 µL: 0.28, 8.4 and 1.3 pmol/vitreous, for dopamine, DOPAC and L-DOPA, respectively.

**MRM Parameters for Blood Samples**

Optimised multiple reaction monitoring (MRM) parameters are summarised in Table S-8B. The corresponding molecular ion and up to three most predominant fragment ions were utilised for each analyte; DOPAC and HVA (and their corresponding deuterated standards) had one MRM transition monitored respectively due to the lack of any additional sufficiently intense fragment ions. Additional MS parameters were as follows: gas temperature and flow rate 340 ^o^C and 8.5 L/min (time segment 1 and 3), 325 ^o^C and 10 L/min (time segment 2), nebuliser 25 psi (time segment 1), and 50 psi (time segment 2 and 3), capillary voltage 3000 V (positive) and 3500 V (negative), cell acceleration voltage 7 V (except HVA and HVA-d_5_ where 2 V was applied). Both quadrupoles were operated in unit resolution.

The limit of quantification (LOQ) of each analyte in the blood matrix was estimated based on a signal to noise ratio of 10:1 for the deuterated quantifier MRM transition and an injection volume of 20 µL: 5.3, 1300 and 170 ng/mL, for dopamine, DOPAC and L-DOPA, respectively.

## Electroretinogram (ERG) measurements

ERG experiments were performed with a purpose-built full-field LED flash system consisting of an LED array, Ganzfeld dome, animal platform, stimulus controlling box and PC-based software (courtesy of the Research School of Physics and Engineering (RSPE), Australian National University (ANU)) and recorded on a 26T Powerlab with LabChart software (AD Instruments, Dunedin, New Zealand). Mice were dark-adapted overnight and anesthetised by intraperitoneal injection of a mixture of ketamine (80 mg/kg) and xylazine (8 mg/kg). Animal temperature was maintained at 37°C by a heated blanket and feedback system linked to a T-type thermocouple rectal probe (AD Instruments, Dunedin, New Zealand; built courtesy of RSPE, ANU). Pupils were dilated with tropicamide (1% w/v, 1 drop (40µL)) and atropine (1% w/v, 1 drop (40µL)) eye drops 10 minutes before the commencement of measurements. Platinum wire loop electrodes were positioned on the surface of the cornea, with stainless steel needle electrodes applied subcutaneously at the middle of the forehead region and the rear leg as reference and ground electrodes, respectively. Single-flash ERGs were obtained under dark-adapted conditions, with single white-flash stimuli produced over 5 steps of illumination (-2, -1.1, -0.2, 0.6 and 1.4 log cd.s/m^2^). Dark adapted responses at each light intensity were averaged from 5-10 recordings, with inter-stimulus intervals ranging from 4s (at -2 log cd.s/m^2^) to 40s (at 1.4 log cd.s/m^2^), with ERG responses showing full recovery between flashes. For light adapted responses, mice were adapted for 10 minutes at 20 cd/m^2^ intensity and then exposed to a series of 0.6 log cd s/m^2^ flashes.

Band-pass filter frequencies were 0.3 and 500 Hz for standard recordings and 75 and 500 Hz to isolate oscillatory potentials which were in turn measured by analysing the amplitude and latency of the second oscillatory potential peak (oscillatory potential 2 (OP2)), which has been shown to be highly correlated with the absolute intensity of the light stimulus.

# Supplementary Figures

**
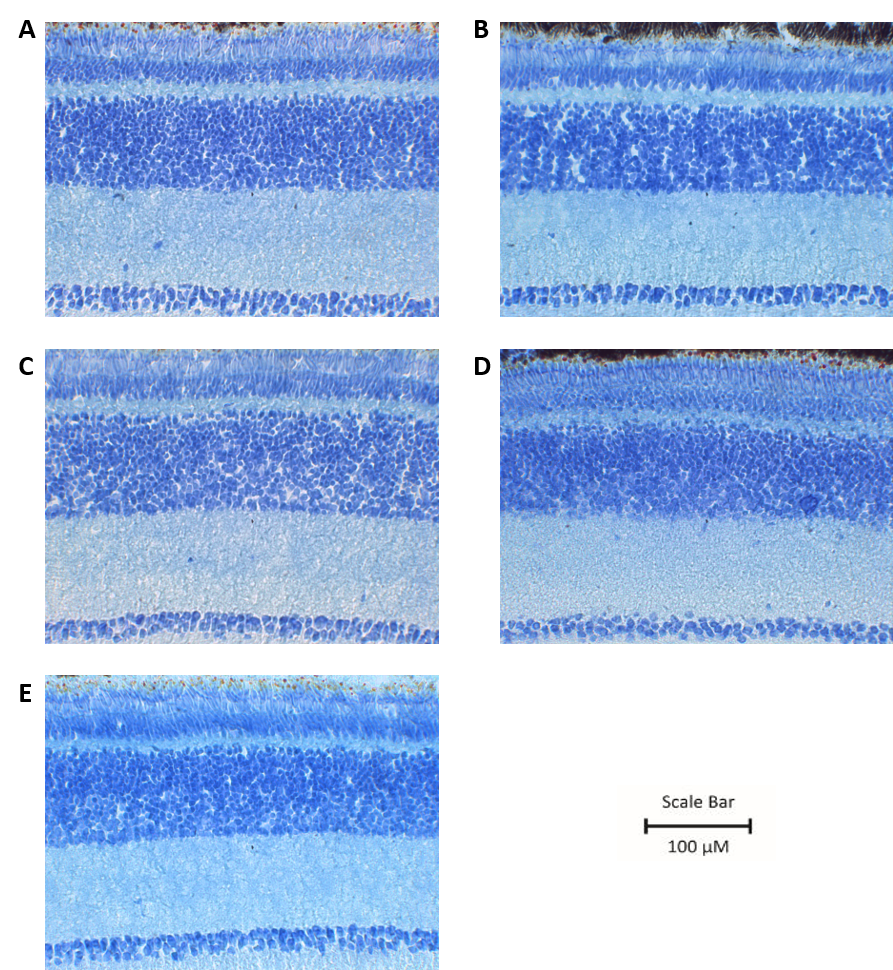
**

**Figure S-1: Retinal histology following long-term (four weeks) treatment.** Chick retinae were stained with 1% w/v toluidine blue solution visualised using 20x magnification. **(A)** Age-matched untreated control; **(B)** FD only eyes (no topical treatment); **(C)** FD 15mM levodopa drops; **(D)** FD 15mM levodopa:3.75mM carbidopa drops; **(E)** FD 15mM levodopa drops in BAK. FD: Form deprivation. BAK: benzalkonium chloride

**
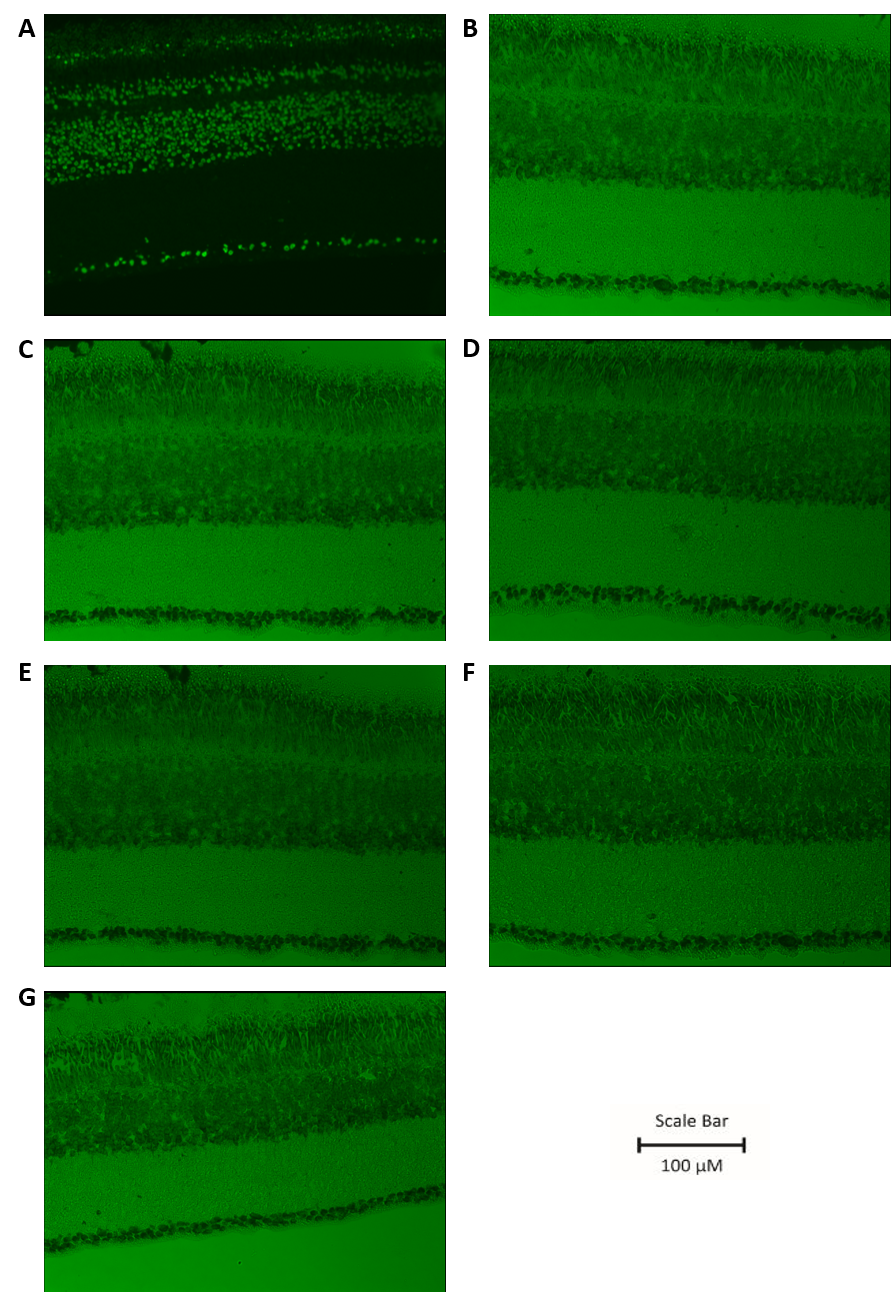
**

**Figure S-2: Fluorescent visualisation of TUNEL staining following long-term (four weeks) treatment.** Chick retinae are visualised using 20x magnification **(A)** Positive control (DNase I treatment was undertaken to simulate the DNA strand breaks characteristic of apoptosis); **(B)** Negative control (no enzyme); **(C)** Age-matched untreated control; **(D)** FD only eyes; **(E)** FD 15mM levodopa drops; **(F)** FD 15mM levodopa:3.75mM carbidopa drops; **(G)** FD 15mM levodopa drops in BAK. FD: Form deprivation, BAK: benzalkonium chloride. Panels B-G were overexposed to visualise retinae as no fluorescence was observed.


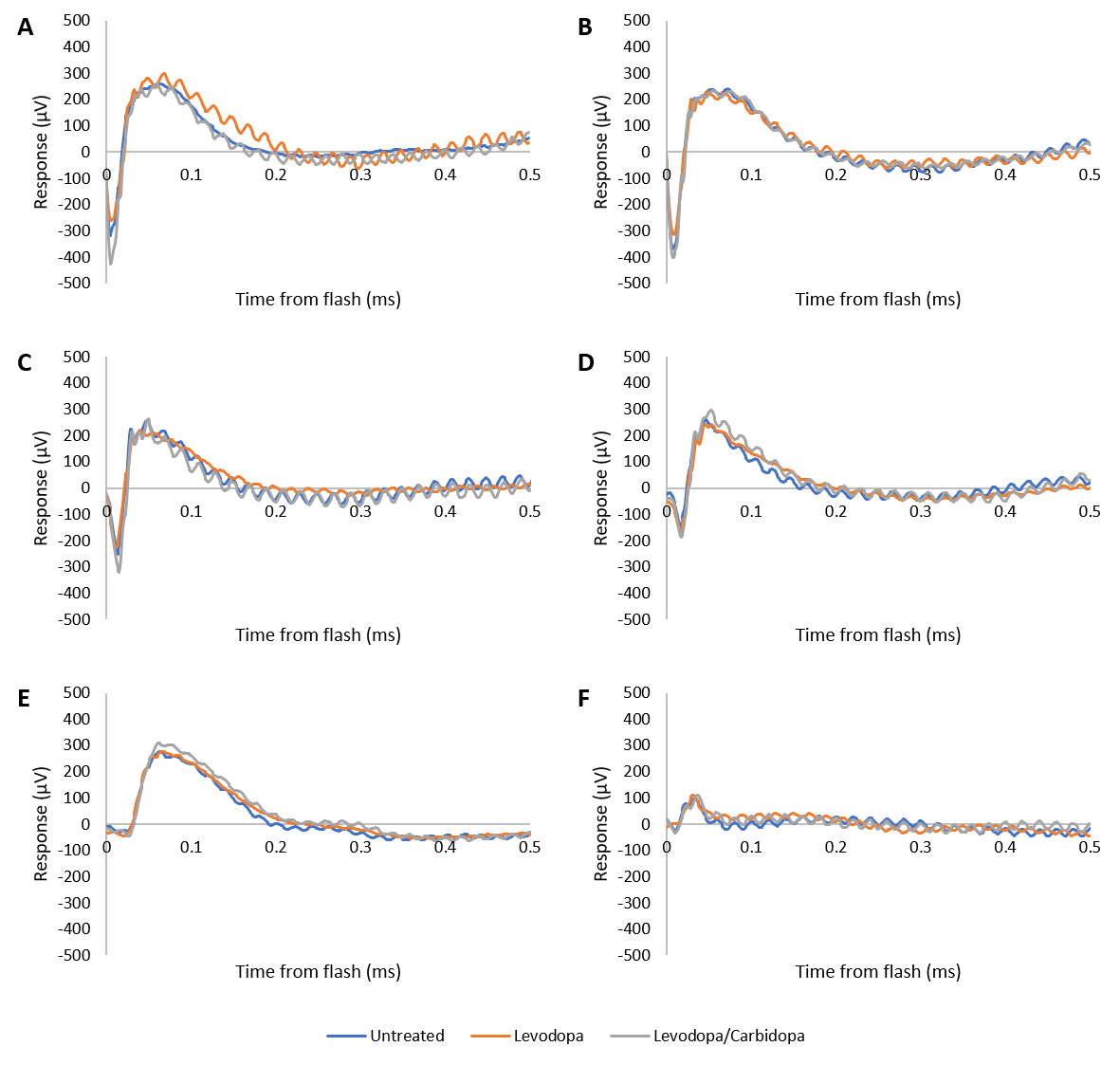


**Figure S-3: Overlay of average ERG responses to chronic (9-month) levodopa topical treatment, levodopa/carbidopa topical treatment, as well as age-matched untreated animals across all light intensities tested.** **(A)** 1.4 log cd.s/m^2^; **(B)** 0.6 log cd.s/m^2^; **(C)** -0.2 log cd.s/m^2^; **(D)** -1.1 log cd.s/m^2^; **(E)** -2 log cd.s/m^2^; **(F)** Light adapted 0.6 log cd.s/m^2^.


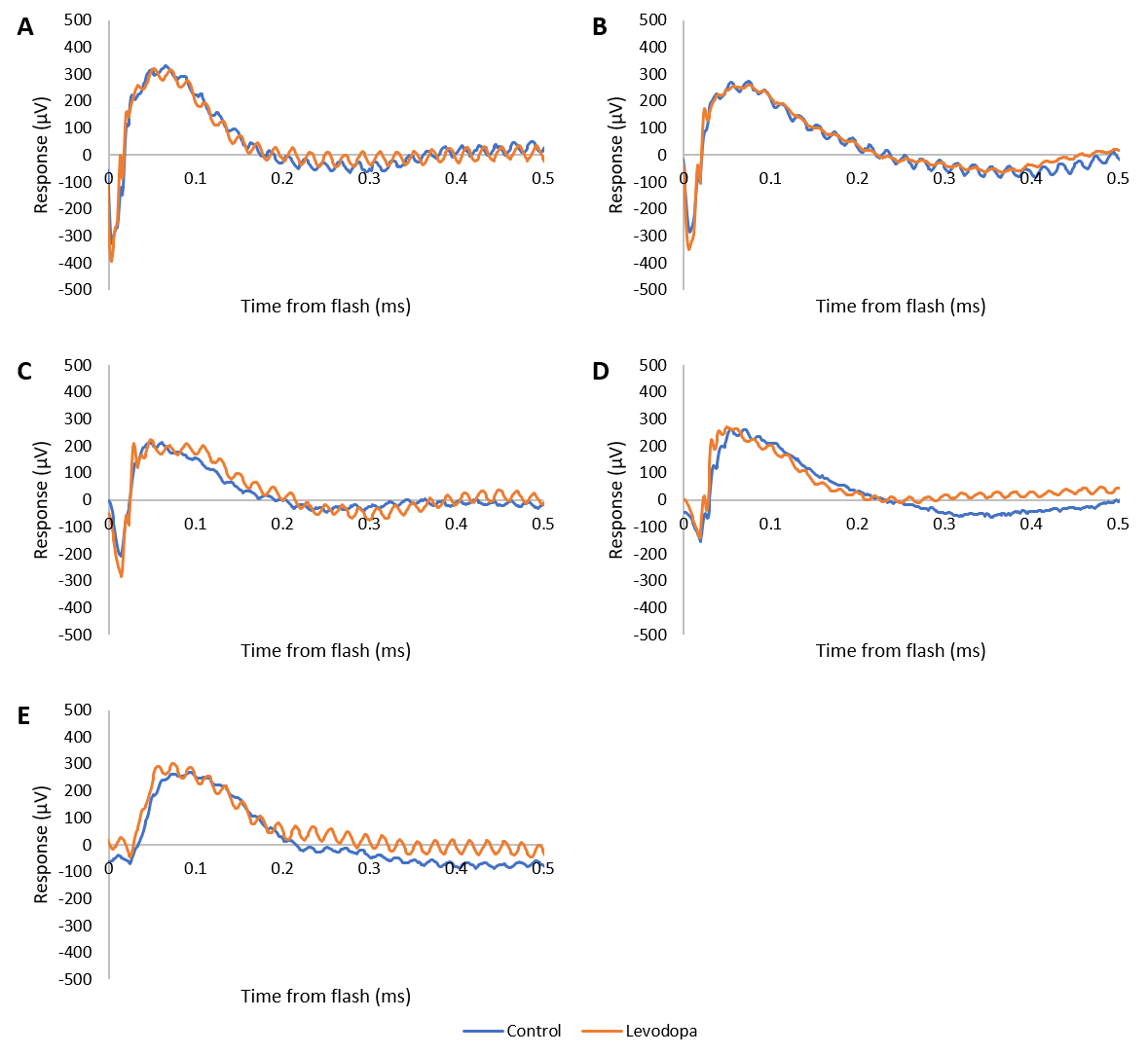


**Figure S-4: Overlay of average ERG response for levodopa and age-matched untreated animals across five light intensities.** ERG measurements were undertaken two hours after topical levodopa treatment. **(A)** 1.4 log cd.s/m^2^; **(B)** 0.6 log cd.s/m^2^; **(C)** -0.2 log cd.s/m^2^; **(D)** -1.1 log cd.s/m^2^; **(E)** -2 log cd.s/m^2^.


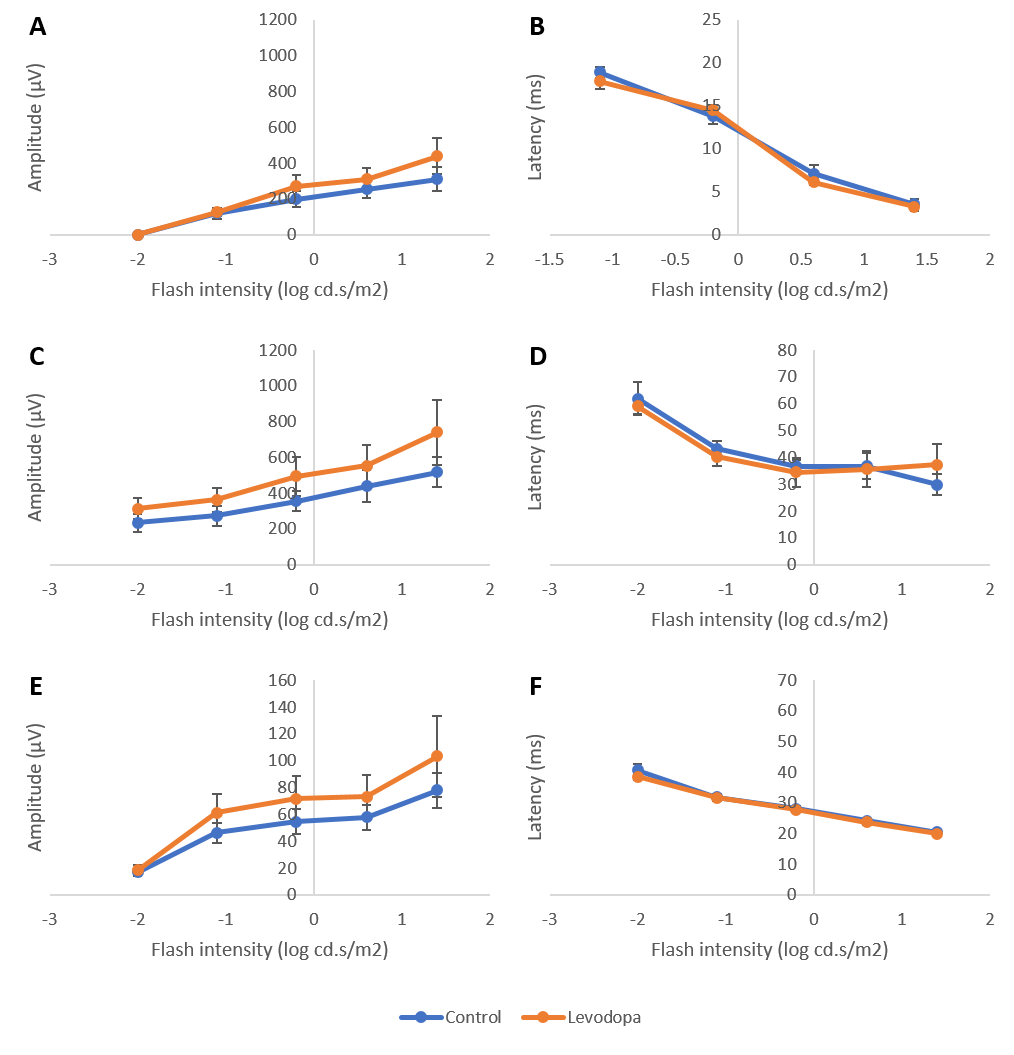


**Figure S-5:** **Effects of topical levodopa on mouse electroretinogram responses two hours post treatment.** No significant difference was observed between control and levodopa treated eyes in **(A)** A-wave amplitude (Wilks’ Lambda=0.783, F(1,9)=0.830, p=0.531); **(B)** A-wave latency (Wilks’ Lambda=0.487, F(1,9)=3.166, p=0.054); **(C)** B-wave amplitude (Wilks’ Lambda=0.728, F(1,9)=0.824, p=0.558); **(D)** B-wave latency (Wilks’ Lambda=0.598, F(1,9)=1.477, p=0.273); **(E)** Oscillatory potential 2 amplitude (Wilks’ Lambda=0.758, F(1,9)=0.704, p=0.632); **(F)** Oscillatory potential 2 latency (Wilks’ Lambda=0.758, F(1,9)=0.704, p=0.632). Data represents mean ± standard error of the mean.

# Supplementary Tables

**Table S-1A: Drug solution formulation and dosages for intravitreal injections of levodopa** (note. mg/kg/day doses are based off an average chick weight of 125g)

| **Drug** | **Application Avenue** | **Ocular Treatment** | **Concentration (mM)** | **Concentration (% w/v)** | **Treatments per Day** | **Volume Given daily (µL)** | **Amount Given (mg/day)** | **Amount Given (mg/kg/day)** | **Number of Animals - 4 Days** | **Number of Animals - 4 Weeks** |
| --- | --- | --- | --- | --- | --- | --- | --- | --- | --- | --- |
| Vehicle Solution | Injection | Diffuser | n/a | n/a | 1 | 10 | n/a | n/a | 10 | - |
| Levodopa | Injection | Diffuser | 0.15 | 0.003 | 1 | 10 | 0.0003 | 0.002 | 8 | - |
| Levodopa | Injection | Diffuser | 1.5 | 0.03 | 1 | 10 | 0.003 | 0.024 | 15 | - |
| Levodopa | Injection | Diffuser | 15 | 0.296 | 1 | 10 | 0.03 | 0.237 | 15 | - |
| Levodopa | Injection | Untreated | 15 | 0.296 | 1 | 10 | 0.03 | 0.237 | 10 | - |
| Levodopa | Injection | Diffuser | 75 | 1.479 | 1 | 10 | 0.148 | 1.183 | 8 | - |

**Table S-1B: Drug solution formulation and dosages for topical eye drops of levodopa**

| **Drug** | **Application Avenue** | **Ocular Treatment** | **Concentration (mM)** | **Concentration (% w/v)** | **Treatments per Day** | **Volume Given daily (µL)** | **Amount Given (mg/day)** | **Amount Given (mg/kg/day)** | **Number of Animals - 4 Days** | **Number of Animals - 4 Weeks** |
| --- | --- | --- | --- | --- | --- | --- | --- | --- | --- | --- |
| Vehicle Solution | Drops | Diffuser | n/a | n/a | 2 | 160 | n/a | n/a | 11 | - |
| Levodopa | Drops | Diffuser | 0.15 | 0.003 | 2 | 160 | 0.005 | 0.038 | 18 | - |
| Levodopa | Drops | Diffuser | 1.5 | 0.03 | 2 | 160 | 0.047 | 0.379 | 18 | - |
| Levodopa | Drops | Diffuser | 15 | 0.296 | 2 | 160 | 0.473 | 3.786 | 14 | 18 |
| Levodopa/Carbidopa | Drops | Diffuser | 15/3.75 | 0.296/0.085 | 2 | 160 | 0.473/0.136 | 3.784/1.088 | 14 | 12 |
| Levodopa/DMSO^*^ | Drops | Diffuser | 15/1410 | 0.296/10 | 2 | 160 | 0.473/17.63 | 3.784/141.01 | 9 | - |
| Levodopa/BAK^#^ | Drops | Diffuser | 15/0.91 | 0.296/0.1 | 2 | 160 | 0.473/0.161 | 3.784/1.286 | - | 6 |
| Levodopa | Drops | Untreated | 15 | 0.296 | 2 | 160 | 0.473 | 3.786 | 15 | - |
| Levodopa | Drops | Diffuser | 45 | 0.887 | 2 | 160 | 1.42 | 11.358 | 9 | - |

*Dimethyl sulfoxide, #Benzalkonium Chloride

**Table S-1C: Drug solution formulation and dosages for intravitreal injections of atropine**

| **Drug** | **Application Avenue** | **Ocular Treatment** | **Concentration (mM)** | **Concentration (% w/v)** | **Treatments per Day** | **Volume Given daily (µL)** | **Amount Given (mg/day)** | **Amount Given (mg/kg/day)** | **Number of Animals - 4 Days** | **Number of Animals - 4 Weeks** |
| --- | --- | --- | --- | --- | --- | --- | --- | --- | --- | --- |
| Atropine | Injection | Diffuser | 0.0015 | 0.0001 | 1 | 10 | 0.0000 | 0.00008336 | 12 | - |
| Atropine | Injection | Diffuser | 0.015 | 0.001 | 1 | 10 | 0.0001 | 0.0008336 | 12 | - |
| Atropine | Injection | Diffuser | 0.15 | 0.01 | 1 | 10 | 0.0010 | 0.008336 | 12 | - |
| Atropine | Injection | Diffuser | 1.5 | 0.1 | 1 | 10 | 0.0104 | 0.0832 | 12 | - |
| Atropine | Injection | Diffuser | 15 | 1 | 1 | 10 | 0.1042 | 0.8336 | 12 | - |

**Table S-1D: Drug solution formulation and dosages for topical eye drops of atropine.**

| **Drug** | **Application Avenue** | **Ocular Treatment** | **Concentration (mM)** | **Concentration (% w/v)** | **Treatments per Day** | **Volume Given daily (µL)** | **Amount Given (mg/day)** | **Amount Given (mg/kg/day)** | **Number of Animals - 4 Days** | **Number of Animals - 4 Weeks** |
| --- | --- | --- | --- | --- | --- | --- | --- | --- | --- | --- |
| Atropine | Drops | Diffuser | 0.015 | 0.001 | 2 | 160 | 0.001667 | 0.013336 | 6 | - |
| Atropine | Drops | Diffuser | 0.15 | 0.01 | 2 | 160 | 0.01667 | 0.13336 | 6 | - |
| Atropine | Drops | Diffuser | 1.5 | 0.1 | 2 | 160 | 0.1667 | 1.3336 | 6 | - |
| Atropine | Drops | Diffuser | 15 | 1 | 2 | 160 | 1.667 | 13.336 | 6 | - |

**Table S-1E: Drug solution formulation and dosages for mouse experiments** (note. mg/kg/day doses are based off final mouse weights of approx. 40g).

| **Drug** | **Application Avenue** | **Concentration (mM)** | **Concentration (% w/v)** | **Treatments per Day** | **Volume Given (µL, daily)** | **Amount Given (mg/day)** | **Amount Given (mg/kg/day)** | **Number of Animals** |
| --- | --- | --- | --- | --- | --- | --- | --- | --- |
| Levodopa | Drops | 15 | 0.296 | 1 | 20 (10 per eye) | 0.059 | 1.479 | 10 |
| Levodopa/Carbidopa | Drops | 15/3.75 | 0.296/0.085 | 1 | 20 (10 per eye) | 0.059/0.017 | 1.479/0.424 | 10 |

**Table S-2: Ultrasonography and refractive measurements (average ± standard error) for chickens treated via intravitreal injection**.

|  |  | **A-Scan Ultrasonography Measurements (mm)** | | | | | **Refraction (D)** | |
| --- | --- | --- | --- | --- | --- | --- | --- | --- |
| **Condition** | **Animal Numbers** | **Anterior Chamber Depth** | **Lens Thickness** | **Vitreal Chamber Depth** | **Axial Length** | **Difference in Axial Length** | **Absolute** | **Difference in Refraction** |
| Form-deprived (FD) Only | 26 | 1.41 ± 0.01 | 2.28 ± 0.01 | 5.40 ± 0.03 | 9.09 ± 0.03 |  | -1.13 ± 0.11 |  |
| FD Only - Contralateral control |  | 1.40 ± 0.01 | 2.27 ± 0.01 | 5.05 ± 0.02 | 8.72 ± 0.02 | 0.37 ± 0.02 | 2.66 ± 0.08 | -3.79 ± 0.11 |
|  |  |  |  |  |  |  |  |  |
| Age-matched Untreated (Left) | 17 | 1.41 ± 0.02 | 2.27 ± 0.02 | 5.02 ± 0.04 | 8.70 ± 0.04 |  | 2.79 ± 0.08 |  |
| Age-matched Untreated (Right) |  | 1.41 ± 0.02 | 2.27 ± 0.02 | 5.04 ± 0.04 | 8.72 ± 0.04 | -0.02 ± 0.01 | 2.78 ± 0.10 | 0.1 ± 0.22 |
|  |  |  |  |  |  |  |  |  |
| FD - 0.15mM Injections | 8 | 1.39 ± 0.02 | 2.27 ± 0.03 | 5.19 ± 0.04 | 8.85 ± 0.04 |  | 0.00 ± 0.29 |  |
| FD - 0.15mM Injections Contra* |  | 1.39 ± 0.02 | 2.29 ± 0.02 | 5.06 ± 0.03 | 8.74 ± 0.03 | 0.11 ± 0.06 | 2.75 ± 0.08 | -2.75 ± 0.37 |
| FD - 1.5mM Injections | 15 | 1.37 ± 0.02 | 2.28 ± 0.02 | 5.16 ± 0.06 | 8.81 ± 0.06 |  | 0.38 ± 0.25 |  |
| FD - 1.5mM Injections Contra * |  | 1.39 ± 0.01 | 2.28 ± 0.02 | 5.05 ± 0.04 | 8.69 ± 0.04 | 0.12 ± 0.04 | 2.92 ± 0.06 | -2.54 ± 0.37 |
| FD - 15mM Injections | 15 | 1.39 ± 0.02 | 2.30 ± 0.02 | 5.07 ± 0.05 | 8.76 ± 0.05 |  | 1.04 ± 0.23 |  |
| FD - 15mM Injections Contra* |  | 1.43 ± 0.02 | 2.28 ± 0.03 | 4.99 ± 0.05 | 8.70 ± 0.05 | 0.06 ± 0.02 | 2.71 ± 0.11 | -1.67 ± 0.26 |
| FD - 75mM injections | 8 | 1.38 ± 0.02 | 2.26 ± 0.03 | 5.00 ± 0.07 | 8.64 ± 0.07 |  | 1.41 ± 0.20 |  |
| FD - 75mM Injections Contra* |  | 1.38 ± 0.02 | 2.27 ± 0.03 | 5.00 ± 0.06 | 8.66 ± 0.06 | -0.02 ± 0.05 | 2.69 ± 0.13 | -1.28 ± 0.34 |
|  |  |  |  |  |  |  |  |  |
| No FD - 15mM Injections | 10 | 1.39 ± 0.02 | 2.30 ± 0.02 | 5.03 ± 0.04 | 8.72 ± 0.04 |  | 2.16 ± 0.17 |  |
| No FD - 15mM Injections Contra* |  | 1.39 ± 0.02 | 2.27 ± 0.02 | 5.07 ± 0.04 | 8.73 ± 0.04 | -0.01 ± 0.02 | 2.82 ± 0.07 | -0.66 ± 0.09 |
| FD - Vehicle Injections | 10 | 1.45 ± 0.02 | 2.23 ± 0.02 | 5.34 ± 0.08 | 9.02 ± 0.08 |  | -0.48 ± 0.18 |  |
| FD - Vehicle Injections Contra* |  | 1.41 ± 0.02 | 2.30 ± 0.02 | 5.04 ± 0.05 | 8.75 ± 0.05 | 0.27 ± 0.06 | 2.34 ± 0.20 | -2.82 ± 0.45 |

*Contralateral control eye, FD: Form deprivation.

**Table S-3: Ultrasonography and refractive measurements (mean ± standard error) for chickens treated via topical eye drops**.

|  |  | **A-Scan Ultrasonography Measurements (mm)** | | | | | **Refraction (D)** | |
| --- | --- | --- | --- | --- | --- | --- | --- | --- |
| **Condition** | **Number of Animals** | **Anterior Chamber Depth** | **Lens Thickness** | **Vitreal Chamber Depth** | **Axial Length** | **Difference in Axial Length** | **Absolute** | **Difference in Refraction** |
| Form-deprived (FD) Only | 26 | 1.41 ± 0.01 | 2.28 ± 0.01 | 5.40 ± 0.03 | 9.09 ± 0.03 |  | -1.13 ± 0.11 |  |
| FD Only - Contralateral control |  | 1.40 ± 0.01 | 2.27 ± 0.01 | 5.05 ± 0.02 | 8.72 ± 0.02 | 0.37 ± 0.02 | 2.66 ± 0.08 | -3.79 ± 0.11 |
|  |  |  |  |  |  |  |  |  |
| Age-matched Untreated (Left) | 17 | 1.41 ± 0.02 | 2.27 ± 0.02 | 5.02 ± 0.04 | 8.70 ± 0.04 |  | 2.79 ± 0.08 |  |
| Age-matched Untreated (Right) |  | 1.41 ± 0.02 | 2.27 ± 0.02 | 5.04 ± 0.04 | 8.72 ± 0.04 | -0.02 ± 0.01 | 2.78 ± 0.10 | 0.1 ± 0.22 |
|  |  |  |  |  |  |  |  |  |
| FD - 0.15mM Drops | 18 | 1.38 ± 0.02 | 2.30 ± 0.02 | 5.29 ± 0.05 | 8.97 ± 0.05 |  | -0.56 ± 0.14 |  |
| FD - 0.15mM Drops Contra* |  | 1.39 ± 0.02 | 2.29 ± 0.02 | 5.03 ± 0.03 | 8.71 ± 0.03 | 0.26 ± 0.04 | 2.77 ± 0.10 | -3.33 ± 0.18 |
| FD - 1.5mM Drops | 18 | 1.38 ± 0.01 | 2.28 ± 0.02 | 5.24 ± 0.05 | 8.90 ± 0.05 |  | -0.13 ± 0.06 |  |
| FD - 1.5mM Drops Contra* |  | 1.35 ± 0.01 | 2.31 ± 0.02 | 5.02 ± 0.03 | 8.68 ± 0.03 | 0.22 ± 0.04 | 3.03 ± 0.05 | -3.16 ± 0.06 |
| FD - 15mM Drops | 14 | 1.42 ± 0.02 | 2.27 ± 0.03 | 5.16 ± 0.06 | 8.85 ± 0.06 |  | 0.15 ± 0.23 |  |
| FD - 15mM Drops Contra* |  | 1.43 ± 0.02 | 2.30 ± 0.02 | 5.00 ± 0.05 | 8.73 ± 0.05 | 0.12 ± 0.04 | 2.48 ± 0.19 | -2.33 ± 0.26 |
| FD - 45mM Drops | 9 | 1.4 ± 0.02 | 2.31 ± 0.02 | 5.11 ± 0.08 | 8.82 ± 0.08 |  | 0.71 ± 0.14 |  |
| FD - 45mM Drops Contra* |  | 1.37 ± 0.01 | 2.3 ± 0.02 | 5.04 ± 0.04 | 8.71 ± 0.04 | 0.11 ± 0.07 | 2.68 ± 0.10 | -1.97 ± 0.18 |
|  |  |  |  |  |  |  |  |  |
| No FD - 15mM Drops Contra* | 15 | 1.40 ± 0.02 | 2.30 ± 0.02 | 5.06 ± 0.04 | 8.76 ± 0.04 |  | 2.49 ± 0.14 |  |
| No FD - 15mM Drops Contra* |  | 1.41 ± 0.02 | 2.30 ± 0.02 | 5.07 ± 0.04 | 8.78 ± 0.04 | -0.02 ± 0.01 | 2.85 ± 0.10 | -0.36 ± 0.05 |
| FD - Vehicle Drops | 11 | 1.43 ± 0.02 | 2.32 ± 0.01 | 5.45 ± 0.07 | 9.20 ± 0.07 |  | -0.73 ± 0.28 |  |
| FD - Vehicle Drops Contra* |  | 1.44 ± 0.02 | 2.26 ± 0.02 | 5.19 ± 0.05 | 8.89 ± 0.04 | 0.31 ± 0.05 | 2.76 ± 0.07 | -3.49 ± 0.37 |
|  |  |  |  |  |  |  |  |  |
| FD - 15mM Drops with Carbidopa | 14 | 1.37 ± 0.02 | 2.24 ± 0.02 | 5.12 ± 0.07 | 8.73 ± 0.07 |  | 0.66 ± 0.30 |  |
| FD - 15mM Drops with Carbidopa Contra* |  | 1.37 ± 0.02 | 2.26 ± 0.02 | 4.99 ± 0.03 | 8.62 ± 0.03 | 0.11 ± 0.05 | 2.60 ± 0.15 | -1.94 ± 0.27 |
| FD - 15mM Drops in DMSO^#^ | 9 | 1.35 ± 0.01 | 2.24 ± 0.04 | 5.18 ± 0.11 | 8.77 ± 0.11 |  | 0.09 ± 0.14 |  |
| FD - 15mM Drops in DMSO^#^ Contra* |  | 1.36 ± 0.00 | 2.27 ± 0.04 | 4.95 ± 0.06 | 8.58 ± 0.06 | 0.19 ± 0.07 | 2.40 ± 0.16 | -2.31 ± 0.20 |

*Contralateral control eye, ^#^dimethyl sulfoxide, FD: Form deprivation

**Table S-4: Long-term A-scan ultrasonography measurements (mean ± standard error, mm)**.

|  |  | **Untreated (Left)** | **Untreated (Right)** | **FD** | **FD Contra** | **FD Levodopa** | **Levodopa Contra** | **FD Levodopa/ Carbidopa** | **Levodopa/ Carbidopa Contra** | **FD Levodopa/ BAK*** | **Levodopa/ BAK* Contra** |
| --- | --- | --- | --- | --- | --- | --- | --- | --- | --- | --- | --- |
| **Before treatment** | **ACD** | 1.29 ± 0.04 | 1.29 ± 0.04 | 1.28 ± 0.02 | 1.28 ± 0.02 | 1.25 ± 0.02 | 1.26 ± 0.02 | 1.26 ± 0.02 | 1.26 ± 0.02 | 1.26 ± 0.04 | 1.26 ± 0.04 |
|  | **Lens** | 2.18 ± 0 | 2.18 ± 0 | 2.11 ± 0.02 | 2.11 ± 0.02 | 2.14 ± 0.02 | 2.14 ± 0.02 | 2.15 ± 0.04 | 2.13 ± 0.03 | 2.18 ± 0 | 2.18 ± 0 |
|  | **VCD** | 4.87 ± 0.03 | 4.87 ± 0.03 | 4.83 ± 0.06 | 4.85 ± 0.06 | 4.85 ± 0.05 | 4.82 ± 0.04 | 4.80 ± 0.08 | 4.82 ± 0.08 | 4.90 ± 0.05 | 4.90 ± 0.04 |
|  | **AL** | 8.34 ± 0.03 | 8.34 ± 0.03 | 8.22 ± 0.06 | 8.23 ± 0.06 | 8.24 ± 0.05 | 8.22 ± 0.04 | 8.20 ± 0.08 | 8.20 ± 0.08 | 8.34 ± 0.05 | 8.32 ± 0.04 |
|  | **Difference in AL** |  | 0 ± 0 |  | -0.01 ± 0.01 |  | 0.02 ± 0.02 |  | 0 ± 0 |  | 0.02 ± 0.02 |
|  |  |  |  |  |  |  |  |  |  |  |  |
| **Week 1** | **ACD** | 1.47 ± 0.02 | 1.47 ± 0.02 | 1.44 ± 0.02 | 1.44 ± 0.02 | 1.45 ± 0.03 | 1.45 ± 0.03 | 1.45 ± 0.02 | 1.47 ± 0.02 | 1.45 ± 0.02 | 1.45 ± 0.02 |
|  | **Lens** | 2.33 ± 0 | 2.33 ± 0 | 2.31 ± 0.01 | 2.32 ± 0.01 | 2.35 ± 0.02 | 2.33 ± 0 | 2.35 ± 0.02 | 2.33 ± 0 | 2.33 ± 0 | 2.33 ± 0 |
|  | **VCD** | 5.08 ± 0.03 | 5.08 ± 0.03 | 5.71 ± 0.08 | 5.05 ± 0.05 | 5.37 ± 0.14 | 4.88 ± 0.07 | 5.40 ± 0.09 | 4.88 ± 0.08 | 5.43 ± 0.11 | 4.90 ± 0.05 |
|  | **AL** | 8.88 ± 0.06 | 8.88 ± 0.06 | 9.46 ± 0.07 | 8.81 ± 0.04 | 9.17 ± 0.12 | 8.65 ± 0.06 | 9.20 ± 0.11 | 8.68 ± 0.07 | 9.21 ± 0.09 | 8.68 ± 0.05 |
|  | **Difference in AL** |  | 0.00 ± 0.01 |  | 0.65 ± 0.06 |  | 0.52 ± 0.08 |  | 0.52 ± 0.07 |  | 0.53 ± 0.07 |
|  |  |  |  |  |  |  |  |  |  |  |  |
| **Week 2** | **ACD** | 1.62 ± 0.01 | 1.62 ± 0.01 | 1.64 ± 0.03 | 1.59 ± 0.02 | 1.58 ± 0.03 | 1.54 ± 0.03 | 1.49 ± 0.03 | 1.55 ± 0.03 | 1.51 ± 0.02 | 1.53 ± 0.03 |
|  | **Lens** | 2.58 ± 0.05 | 2.58 ± 0.05 | 2.54 ± 0.02 | 2.54 ± 0.04 | 2.55 ± 0.03 | 2.50 ± 0.03 | 2.53 ± 0.03 | 2.49 ± 0.02 | 2.49 ± 0.02 | 2.49 ± 0.02 |
|  | **VCD** | 5.33 ± 0.06 | 5.33 ± 0.06 | 6.37 ± 0.08 | 5.16 ± 0.09 | 6.09 ± 0.20 | 5.16 ± 0.07 | 6.06 ± 0.18 | 5.11 ± 0.12 | 6.19 ± 0.13 | 5.17 ± 0.04 |
|  | **AL** | 9.52 ± 0.09 | 9.52 ± 0.09 | 10.55 ± 0.09 | 9.29 ± 0.08 | 10.22 ± 0.19 | 9.19 ± 0.06 | 10.09 ± 0.17 | 9.16 ± 0.11 | 10.20 ± 0.13 | 9.19 ± 0.04 |
|  | **Difference in AL** |  | 0.00 ± 0 |  | 1.26 ± 0.10 |  | 1.03 ± 0.14 |  | 0.93 ± 0.12 |  | 1.00 ± 0.11 |
|  |  |  |  |  |  |  |  |  |  |  |  |
| **Week 3** | **ACD** | 1.77 ± 0 | 1.77 ± 0 | 1.87 ± 0.04 | 1.72 ± 0.02 | 1.81 ± 0.03 | 1.71 ± 0.02 | 1.79 ± 0.05 | 1.73 ± 0.04 | 1.81 ± 0.04 | 1.73 ± 0.03 |
|  | **Lens** | 2.74 ± 0.02 | 2.74 ± 0.02 | 2.73 ± 0.02 | 2.71 ± 0.02 | 2.72 ± 0.02 | 2.70 ± 0.02 | 2.66 ± 0.03 | 2.68 ± 0.03 | 2.73 ± 0.03 | 2.75 ± 0.02 |
|  | **VCD** | 5.64 ± 0.06 | 5.66 ± 0.07 | 6.97 ± 0.13 | 5.61 ± 0.06 | 6.46 ± 0.11 | 5.37 ± 0.07 | 6.47 ± 0.20 | 5.35 ± 0.12 | 6.59 ± 0.20 | 5.43 ± 0.05 |
|  | **AL** | 10.15 ± 0.10 | 10.17 ± 0.12 | 11.57 ± 0.12 | 10.04 ± 0.07 | 11.11 ± 0.10 | 9.82 ± 0.07 | 10.92 ± 0.21 | 9.77 ± 0.13 | 11.13 ± 0.20 | 9.91 ± 0.05 |
|  | **Difference in AL** |  | -0.03 ± 0.02 |  | 1.52 ± 0.14 |  | 1.29 ± 0.12 |  | 1.16 ± 0.20 |  | 1.22 ± 0.18 |
|  |  |  |  |  |  |  |  |  |  |  |  |
| **Week 4** | **ACD** | 1.79 ± 0.03 | 1.77 ± 0.03 | 1.98 ± 0.05 | 1.79 ± 0.03 | 1.88 ± 0.07 | 1.75 ± 0.02 | 1.86 ± 0.04 | 1.75 ± 0.04 | 1.83 ± 0.07 | 1.75 ± 0.02 |
|  | **Lens** | 2.81 ± 0.05 | 2.86 ± 0.03 | 2.82 ± 0.04 | 2.83 ± 0.02 | 2.84 ± 0.03 | 2.75 ± 0.03 | 2.79 ± 0.02 | 2.77 ± 0.00 | 2.77 ± 0.00 | 2.79 ± 0.02 |
|  | **VCD** | 5.98 ± 0.08 | 5.94 ± 0.08 | 7.52 ± 0.20 | 5.87 ± 0.07 | 6.60 ± 0.17 | 5.63 ± 0.07 | 6.52 ± 0.19 | 5.62 ± 0.14 | 6.82 ± 0.29 | 5.60 ± 0.08 |
|  | **AL** | 10.56 ± 0.12 | 10.57 ± 0.13 | 12.32 ± 0.19 | 10.48 ± 0.10 | 11.32 ± 0.22 | 10.12 ± 0.05 | 11.18 ± 0.20 | 10.14 ± 0.12 | 11.41 ± 0.29 | 10.14 ± 0.08 |
|  | **Difference in AL** |  | -0.01 ± 0.03 |  | 1.84 ± 0.19 |  | 1.20 ± 0.21 |  | 1.04 ± 0.23 |  | 1.28 ± 0.23 |

‘Difference in AL’ represents the difference in axial length between the left and right eye of each animal. *Benzalkonium chloride, ACD: Anterior chamber depth, Lens: Lens thickness, VCD: Vitreal chamber depth, AL: Axial Length, FD: Form deprivation

**Table S-5: Long-term refraction measurements (mean ± standard error, D).**

|  |  | **Untreated** | **FD** | **FD Levodopa** | **FD Levodopa/ Carbidopa** | **FD Levodopa/ BAK*** |
| --- | --- | --- | --- | --- | --- | --- |
| **Before treatment** | Left | 2.8 ± 0.04 | 2.68 ± 0.03 | 2.78 ± 0.08 | 2.63 ± 0.07 | 2.70 ± 0.13 |
|  | Right | 2.7 ± 0.03 | 2.73 ± 0.03 | 2.74 ± 0.09 | 2.63 ± 0.07 | 2.65 ± 0.07 |
|  | Difference | 0.1 ± 0.04 | -0.05 ± 0.02 | 0.04 ± 0.05 | 0 ± 0 | 0.05 ± 0.06 |
|  |  |  |  |  |  |  |
| **Week 1** | Left | 2.36 ± 0.08 | -1.71 ± 0.25 | -0.03 ± 0.10 | -0.24 ± 0.18 | -1.36 ± 0.19 |
|  | Right | 2.42 ± 0.09 | 2.41 ± 0.08 | 2.10 ± 0.15 | 1.69 ± 0.13 | 2.59 ± 0.24 |
|  | Difference | -0.05 ± 0.05 | -4.12 ± 0.24 | -2.1 ± 0.15 | -1.93 ± 0.24 | -3.87 ± 0.33 |
|  |  |  |  |  |  |  |
| **Week 2** | Left | 2.04 ± 0.20 | -2.50 ± 0.34 | -0.48 ± 0.19 | -0.20 ± 0.19 | -0.89 ± 0.40 |
|  | Right | 1.94 ± 0.21 | 2.05 ± 0.16 | 2.08 ± 0.27 | 1.97 ± 0.21 | 2.34 ± 0.21 |
|  | Difference | 0.03 ± 0.03 | -4.55 ± 0.39 | -2.57 ± 0.35 | -2.17 ± 0.25 | -3.23 ± 0.49 |
|  |  |  |  |  |  |  |
| **Week 3** | Left | 1.86 ± 0.20 | -2.27 ± 0.14 | -0.33 ± 0.32 | -0.23 ± 0.33 | -0.38 ± 0.30 |
|  | Right | 1.94 ± 0.19 | 1.90 ± 0.21 | 2.35 ± 0.21 | 2.25 ± 0.26 | 2.28 ± 0.20 |
|  | Difference | -0.07 ± 0.05 | -4.17 ± 0.26 | -2.68 ± 0.23 | -2.48 ± 0.49 | -2.67 ± 0.38 |
|  |  |  |  |  |  |  |
| **Week 4** | Left | 1.20 ± 0.21 | -2.88 ± 0.35 | 0.00 ± 0.27 | 0.03 ± 0.17 | -0.88 ± 0.44 |
|  | Right | 1.51 ± 0.20 | 1.68 ± 0.16 | 2.53 ± 0.31 | 2.05 ± 0.35 | 1.97 ± 0.26 |
|  | Difference | -0.11 ± 0.15 | -4.57 ± 0.30 | -2.53 ± 0.31 | -2.02 ± 0.30 | -2.93 ± 0.33 |

‘Difference’ represents the difference in refraction between the left and right eye of each animal. *Benzalkonium chloride, FD: Form deprivation.

**Table S-6: Long-term influence of 15mM levodopa drops on corneal thickness and IOP**.

|  |  | **FD** | **FD Levodopa** | **FD Levodopa/ Carbidopa** | **FD Levodopa/ BAK*** |
| --- | --- | --- | --- | --- | --- |
| **Corneal Thickness (μm)** | **Left** | 218.8 ± 7.55 | 199.4 ± 10.18 | 212.3 ± 6.3 | 195.14 ± 1.58 |
|  | **Right** | 213 ± 3.12 | 200 ± 4.24 | 216.6 ± 8.67 | 208.7 ± 4.51 |
|  |  |  |  |  |  |
| **IOP (mmHg)** | **Left** | 20.51 ± 0.76 | 19.18 ± 0.59 | 19.77 ± 0.69 | 20.83 ± 0.42 |
|  | **Right** | 20.52 ± 0.23 | 20.16 ± 0.25 | 19.43 ± 0.59 | 20.36 ± 0.34 |

*Benzalkonium chloride, FD: Form deprivation

**Table S-7: A-scan ultrasonography and refractive measurements for atropine treated chickens.**

|  |  | **A-Scan Ultrasonography Measurements (mm ± Std Err)** | | | | | **Refraction (D ± Std Err)** | |
| --- | --- | --- | --- | --- | --- | --- | --- | --- |
| **Condition** | **Number of Animals** | **Anterior Chamber Depth** | **Lens Thickness** | **Vitreal Chamber Depth** | **Axial Length** | **Difference in Axial Length** | **Absolute** | **Difference in Refraction** |
| Form-deprived (FD) Only | 26 | 1.41 ± 0.01 | 2.28 ± 0.01 | 5.40 ± 0.03 | 9.09 ± 0.03 |  | -1.13 ± 0.11 |  |
| FD Only - Contralateral control |  | 1.40 ± 0.01 | 2.27 ± 0.01 | 5.05 ± 0.02 | 8.72 ± 0.02 | 0.37 ± 0.02 | 2.66 ± 0.08 | -3.79 ± 0.11 |
|  |  |  |  |  |  |  |  |  |
| Age-matched Untreated (Left) | 17 | 1.41 ± 0.02 | 2.27 ± 0.02 | 5.02 ± 0.04 | 8.70 ± 0.04 |  | 2.79 ± 0.08 |  |
| Age-matched Untreated (Right) |  | 1.41 ± 0.02 | 2.27 ± 0.02 | 5.04 ± 0.04 | 8.72 ± 0.04 | -0.02 ± 0.01 | 2.78 ± 0.10 | 0.1 ± 0.22 |
|  |  |  |  |  |  |  |  |  |
| FD - 0.0015mM Injections | 12 | 1.37 ± 0.02 | 2.28 ± 0.02 | 5.14 ± 0.07 | 8.78 ± 0.08 |  | -0.47 ± 0.17 |  |
| FD - 0.0015mM Injections Contra* |  | 1.40 ± 0.02 | 2.29 ± 0.02 | 4.95 ± 0.05 | 8.63 ± 0.05 | 0.15 ± 0.05 | 2.56 ± 0.08 | -3.03 ± 0.19 |
| FD – 0.015mM Injections | 12 | 1.39 ± 0.01 | 2.27 ± 0.01 | 5.09 ± 0.03 | 8.74 ± 0.03 |  | -0.33 ± 0.15 |  |
| FD – 0.015mM Injections Contra* |  | 1.38 ± 0.01 | 2.27 ± 0.01 | 4.95 ± 0.02 | 8.60 ± 0.02 | 0.14 ± 0.02 | 2.24 ± 0.10 | -2.57 ± 0.16 |
| FD – 0.15mM Injections | 12 | 1.38 ± 0.01 | 2.21 ± 0.02 | 5.13 ± 0.04 | 8.72 ± 0.05 |  | -0.47 ± 0.28 |  |
| FD – 0.15mM Injections Contra* |  | 1.38 ± 0.02 | 2.24 ± 0.02 | 4.99 ± 0.02 | 8.61 ± 0.03 | 0.12 ± 0.04 | 2.03 ± 0.29 | -2.5 ± 0.54 |
| FD – 1.5mM Injections | 12 | 1.40 ± 0.02 | 2.24 ± 0.02 | 5.05 ± 0.03 | 8.68 ± 0.04 |  | 0.1 ± 0.19 |  |
| FD – 1.5mM Injections Contra* |  | 1.37 ± 0.01 | 2.25 ± 0.02 | 4.98 ± 0.02 | 8.60 ± 0.02 | 0.08 ± 0.03 | 2.59 ± 0.09 | -2.49 ± 0.10 |
| FD – 15mM Injections | 12 | 1.37 ± 0.01 | 2.26 ± 0.03 | 4.92 ± 0.07 | 8.55 ± 0.07 |  | 0.07 ± 0.13 |  |
| FD – 15mM Injections Contra* |  | 1.36 ± 0.00 | 2.26 ± 0.02 | 4.94 ± 0.03 | 8.56 ± 0.03 | -0.01 ± 0.05 | 2.34 ± 0.37 | -2.27 ± 0.01 |
|  |  |  |  |  |  |  |  |  |
| FD - 0.015mM Drops | 6 | 1.40 ± 0.02 | 2.28 ±0.03 | 5.16 ± 0.06 | 8.85 ± 0.07 |  | -0.85 ± 0.56 |  |
| FD - 0.015mM Drops Contra* |  | 1.40 ± 0.03 | 2.23 ± 0.03 | 4.98 ± 0.05 | 8.62 ± 0.04 | 0.23 ± 0.04 | 2 ± 0.27 | -2.85 ± 0.57 |
| FD – 0.15mM Drops | 6 | 1.47 ± 0.02 | 2.26 ± 0.03 | 5.16 ± 0.03 | 8.88 ± 0.03 |  | -0.62 ± 0.59 |  |
| FD – 0.15mM Drops Contra* |  | 1.45 ± 0.03 | 2.28 ± 0.03 | 4.95 ± 0.03 | 8.67 ± 0.03 | 0.21 ± 0.04 | 1.9 ± 0.24 | -2.52 ± 0.49 |
| FD – 1.5mM Drops | 6 | 1.39 ± 0.02 | 2.26 ±0.03 | 5.17 ± 0.05 | 8.82 ± 0.06 |  | -0.37 ± 0.22 |  |
| FD – 1.5mM Drops Contra* |  | 1.39 ± 0.02 | 2.27 ± 0.03 | 4.94 ± 0.03 | 8.60 ± 0.03 | 0.22 ± 0.03 | 1.7 ± 0.08 | -2.07 ± 0.19 |
| FD - 15mM Drops | 6 | 1.40 ± 0.03 | 2.18 ± 0.00 | 5.10 ± 0.06 | 8.69 ± 0.06 |  | 0.1 ± 0.49 |  |
| FD - 15mM Drops Contra* |  | 1.43 ± 0.03 | 2.18 ± 0.00 | 4.96 ± 0.04 | 8.56 ± 0.04 | 0.13 ± 0.04 | 2.4 ± 0.29 | -2.3 ± 0.65 |

FD: Form deprivation, Contra: Contralateral control

**Table S-8A: Vitreous Sample MRM transitions** (precursor and product ion(s), with corresponding fragmentor voltage, collision energies, and dwell times for each analyte and internal standard.

| **Analyte/Internal Standard** | **Precursor Ion (m/z)** | **Quantifier transition product ion (*m/z*)** | **Fragmentor Voltage, Collision Energy (volts)** | **Qualifier transition product ion(s) (*m/z*)** | **Fragmentor Voltage, Collision Energy (volts)** | **Dwell time (ms)** |
| --- | --- | --- | --- | --- | --- | --- |
| **Time Segment 1** (positive ion mode) | | | | | | |
| Dopamine | 154.1 | 137.1 | 62, 8 | 91.1 | 60, 28 | 78 |
|  |  |  |  | 65.1 | 60, 40 |  |
| Dopamine-d_4_ | 158.1 | 141.1 | 60, 8 | 95.1 | 60, 28 | 50 |
| Levodopa | 198.1 | 152.2 | 79, 8 | 107.1 | 79, 28 | 78 |
| Levodopa-d_3_ | 201.1 | 155.1 | 72, 8 | 109.1 | 72, 28 | 50 |
| **Time Segment 2** (negative ion mode) | | | | | | |
| DOPAC | 167 | 123.1 | 60, 4 | - | - | 161 |
| DOPAC-d_5_ | 172.1 | 128.1 | 60, 4 | - | - | 161 |

**Table S-8B: Blood Sample MRM transitions** (precursor and product ion(s), with corresponding fragmentor voltage, collision energies, and dwell times for each analyte and internal standard.

| **Analyte/Internal standard** | **Precursor Ion (m/z)** | **Quantifier transition product ion (*m/z*)** | **Fragmentor Voltage, Collision Energy (volts)** | **Qualifier transition product ion(s) (*m/z*)** | **Fragmentor Voltage, Collision Energy (volts)** | **Dwell time (ms)** |
| --- | --- | --- | --- | --- | --- | --- |
|  |  |  |  |  |  |  |
| **Time Segment 1** (positive ion mode) | | | | | | |
| Dopamine | 154.1 | 137.1 | 62, 8 | 91.1 | 60, 28 | 78 |
|  |  |  |  | 65.1 | 60, 40 |  |
| Dopamine-d_4_ | 158.1 | 141.1 | 60, 8 | 95.1 | 60, 28 | 50 |
| Levodopa | 198.1 | 152.2 | 79, 8 | 107.1 | 79, 28 | 78 |
| Levodopa-d_3_ | 201.1 | 155.1 | 72, 8 | 109.1 | 72, 28 | 50 |
| **Time Segment 2** (negative ion mode) | | | | | | |
| DOPAC | 167 | 123.1 | 60, 4 | - | - | 161 |
| DOPAC-d_5_ | 172.1 | 128.1 | 60, 4 | - | - | 161 |
| **Time Segment 3** (negative ion mode) | | | | | | |
| HVA | 181.1 | 137.1 | 60, 4 | - | - | 250 |
| HVA-d_5_ | 186.1 | 142.2 | 60, 4 | - | - | 250 |
